# Physiological and molecular mechanisms of glycine betaine in alleviating Na_2_SO_4_ stress in *Glycyrrhiza uralensis*

**DOI:** 10.3389/fpls.2025.1667006

**Published:** 2025-11-04

**Authors:** Junjun Gu, Xiaomei Ma, Jiatong Liu, Miao Ma

**Affiliations:** College of Life Sciences, Shihezi University, Key Laboratory of Oasis Town and Mountain-basin System Ecology, Key Laboratory of Xinjiang Phytomedicine Resource Utilization, Ministry of Education, Shihezi, China

**Keywords:** licorice, transcriptome, metabolome, salt stress, antioxidants

## Abstract

Salt stress is a common environmental factor that leads to low yield and quality in *Glycyrrhiza uralensis*. Although exogenous foliar application of glycine betaine (GB) can improve salt tolerance, its underlying mechanisms remain unclear. Therefore, this study systematically investigated the effects of GB (0, 10, 20, 40, and 80 mM) on the physiology, transcriptome, and metabolome of *G. uralensis* seedlings subjected to 160 mM Na_2_SO_4_ stress conditions. Results indicate that GB significantly increased endogenous GB levels and Betaine aldehyde dehydrogenase activity in various seedling organs, effectively enhanced the activities of antioxidant enzymes (SOD, CAT, POD, APX) and the concentration of the antioxidant AsA in the roots and leaves. Furthermore, GB application elevated the concentrations of soluble proteins and proline, and boosted the secretion rates of K^+^, Na^+^, and Ca^2+^, while significantly reduced levels of reactive oxygen species (O_2_^-^, H_2_O_2_), malondialdehyde (MDA), and electrolyte leakage. Consequently, seedling biomass increased significantly. Transcriptomics identified 2389 and 3935 differentially expressed genes (DEGs) in leaves at 6 h and 24 h post-GB application, respectively. Metabolomics detected 361 and 617 differential metabolites (DMs) at these time points. At 6 h, GB application significantly activated genes in the zeatin biosynthesis and plant-pathogen interaction pathways, and promoted the accumulation of intermediate metabolites in arachidonic acid metabolism, linoleic acid metabolism, and unsaturated fatty acid biosynthesis. After 24 h, GB upregulated genes in key pathways such as phenylpropanoid biosynthesis and flavonoid biosynthesis. Conversely, GB suppressed the accumulation of intermediates in monoterpene biosynthesis. The combined analysis results indicated that the flavone and flavonol biosynthesis pathways showed a sustained response to GB application under salt stress. In summary, exogenous GB effectively bolsters salt tolerance in *G. uralensis* seedlings by enhancing antioxidant capacity, osmotic regulation, and ion secretion efficiency. Moreover, it stimulates the expression of genes involved in the synthesis of secondary metabolites, carbohydrates, lipids, and hormones. These findings provide novel comprehensive insights into GB-mediated salt tolerance and offer valuable genetic resources and a theoretical foundation for breeding salt-tolerant *G. uralensis* varieties.

## 1 Introduction

Excessive soil salinity is a major constraint on plant growth (Yang and Guo, 2018). It induces osmotic stress and hinders root water uptake (Isayenkov et al., 2020). The accumulation of Na^+^ further disrupts the uptake of other essential nutrients (Muhammad et al., 2017), and triggers a rapid accumulation of intracellular reactive oxygen species (ROS) such as superoxide anions (O_2_^-^), hydrogen peroxide (H_2_O_2_), hydroxyl radicals (·OH) and singlet oxygen (^1^O_2_). Subsequently, ROS leads to increased membrane permeability and electrolyte leakage, ultimately impairing photosynthetic carbon assimilation, respiratory processes, and other core metabolic pathways (Rim et al., 2021). Globally, approximately 932.2 million hectares of arable land are affected by salinization (Hassani et al., 2020). Factors such as strong evaporation, improper irrigation, and excessive fertilization are expected to exacerbate this issue, further expanding salinized farmland (Huang et al., 2021). This trend threatens farmland ecosystems, increasing agricultural costs, and reducing crop yield and quality (Lv et al., 2025).

To mitigate the adverse effects of salt stress on crops, researchers employ exogenous compounds. This strategy aims to bolster short-term salt tolerance, enabling plants to successfully withstand the stress-sensitive phase. Glycine betaine (GB), a chemical compound widely distributed in organisms (Colak et al., 2024; Jin et al., 2024), is extensively utilized in crops due to its roles as an antioxidant, osmotic regulator, and nitrogen source (Li et al., 2025). Research demonstrates that glycine betaine (GB) not only maintains cellular osmotic homeostasis (Islam et al., 2024), but also enhances accumulation of osmoprotectants including proline, soluble sugars, and soluble proteins (Estaji et al., 2019). Concurrently, GB restricts root acquisition of Na^+^ and Cl^-^ while facilitating translocation of K^+^ and Ca²^+^ to aerial tissues (Zuzunaga-Rosas et al., 2022; Habib et al., 2012). Furthermore, it coordinately elevates non-enzymatic antioxidants (glutathione, ascorbate) and augments activities of key antioxidant enzymes—catalase, superoxide dismutase, and ascorbate peroxidase (Khalifa et al., 2016; Shams et al., 2016) —collectively reinforcing salt tolerance. Additionally, GB counteracts salinity stress by promoting carbon/nitrogen metabolism and upregulating phenylpropanoid pathway-associated gene expression (Kim et al., 2020). Although the alleviating effect of GB on salt stress has been widely demonstrated in crops like microalgae (Song et al., 2024), rice (Rahman et al., 2002), and tomatoes (Sajyan et al., 2019), its precise physiological and molecular mechanisms remain unclear.

*Glycyrrhiza uralensis*, a perennial legume, is widely distributed across northern China, Mongolia, the Siberian region of Russia, Kazakhstan, and Pakistan (Christian et al., 2018). It is also favored and extensively utilized in countries such as South Korea, Japan, and the United States (Nose et al., 2017). The dried roots of *G. uralensis* are traditional medicinal materials, known for their cough-suppressing, phlegm-relieving, and asthma-alleviating properties (Kuang et al., 2018). These roots are rich in flavonoids and triterpenoids, which have demonstrated antioxidant, free radical scavenging, antiviral and neuroprotective effects, leading to widespread application in cosmetics (Wang et al., 2022) and pharmaceuticals (Selyutina and Polyakov, 2019). Notably, glycyrrhizin, a natural sweetener, is commonly used to improve the flavor of foods for diabetic patients (Pandey and Ayangla, 2018). However, extensive harvesting and habitat destruction have dramatically reduced wild licorice populations in size and scale. Consequently, cultivated licorice has emerged as a key substitute. Although mature *G. uralensis* plants exhibit strong salt tolerance—enabling saline soil reclamation and economic utilization—their seedlings display marked halotolerance deficiency, severely limiting cultivation in saline-affected areas. Given GB’s established osmoprotective and antioxidant functions, exogenous GB application represents a promising strategy to enhance salinity resilience in *G. uralensis* seedlings, though the underlying mechanisms require further elucidation.

To elucidate these underlying mechanisms, we employed a combined physiological and multi-omics approach. RNA sequencing (RNA-seq) enables rapid, comprehensive profiling of gene expression in seedlings under salt stress, while metabolomics quantifies small-molecule metabolites to elucidate the relationship between GB treatment and salt tolerance. In this study, biomass accumulation, antioxidant activity, osmotic regulation, ion secretion, gene expression, and metabolite profiles were assessed in *G. uralensis* seedlings exposed to salt stress combined with GB treatment. This investigation aims to uncover the physiological and molecular mechanisms underlying exogenous GB-enhanced salt tolerance in *G. uralensis*, providing a scientific foundation for enhancing cultivated licorice resilience in saline soils via GB application.

## 2 Materials and methods

### 2.1 Plant materials

Glycine betaine (GB, molecular weight: 118.15 g/mol, purity >98%) was purchased from McLean Company (Shanghai, China). *G. uralensis* seeds were provided by the Licorice Research Institute of Shihezi University. The experiment was conducted at the Shihezi University campus from April 2021 to October 2021.

### 2.2 Experimental design

Uniform and plump seeds of *G. uralensis* were selected and immersed in 98% H_2_SO^4^ for 30 min, followed by thorough rinsing with distilled water to remove residual acid on the seed surface. After an 8-h hydration in distilled water, the swollen seeds were evenly sown in pots (diameter: bottom 20 cm × top 30 cm × height 20 cm) under a rain shelter, with a total of 30 pots. The substrate comprised a 3:7 (v/v) river sand:loam mixture sterilized with 2% carbendazim. The growth conditions were maintained day/night temperatures of 25–36°C and 18–23°C, respectively, with 50–60% relative humidity. The soil properties were as follows: pH 7.8, total nitrogen, phosphorus, and potassium concentrations were 0.315 g/kg, 0.131 g/kg and 5.47 g/kg, respectively, available nitrogen, phosphorus, and potassium levels were 52.59 mg/kg, 5.23 mg/kg and 50.04 mg/kg, respectively, and the organic matter content was 6.64 mg/kg (Jia et al., 2023). At the four-true-leaf stage, excess seedlings were removed to four uniform plants per pot. After 30 days, salt stress was induced by irrigating with 160 mM Na_2_SO^4^, applying 200 mL per pot every two days for a total of 15 applications (Zhang et al., 2018). The Control group (CK) received the same volume of distilled water. One week following salt treatment, GB solutions (0, 10, 20, 40, and 80 mM) were applied at 200 mL per pot every two days in three applications (Dong et al., 2024), creating six treatment groups: (1) CK (no salt, no GB), (2) S (salt only), (3) S+GB10, (4) S+GB20, (5) S+GB40, and (6) S+GB80 (n=3 pots per group). On the second day after completing the final GB treatment, randomly collect the second fully expanded leaf at the top of the stem from each treatment group for physiological index measurement and optimal concentration screening. The entire experiment was independently repeated three times. For each experimental repetition, each treatment included three biological replicates.

Based on biomass data, the S+GB40 treatment group had a significantly better promoting effect on the biomass of *G. uralensis* roots, stems, and leaves than other GB concentrations. Therefore, 40 mM was selected as the GB working concentration for subsequent experiments. To further analyze the mechanism of GB in alleviating salt stress, blank control (CK), salt stress group (S), and S+GB40 (S+GB) were set up again (n= 3 pots per group). In the S+GB group, leaf samples were collected at 6 h (marked as S+GB+6 h) and 24 h (marked as S+GB+24 h) after GB treatment, and were used for transcriptome and metabolome analysis together with the CK and S group samples collected synchronously.

### 2.3 Analysis of endogenous glycine betaine and BADH2 activity

The content of GB in the roots, stems, and leaves of *G. uralensis* was determined using a glycine betaine assay kit (GB-BC3130, Beijing Solarbio Technology Co., Ltd., China). To assess the capacity for endogenous GB synthesis, we measured the activity of betaine aldehyde dehydrogenase (BADH2), which catalyzes the oxidation of betaine aldehyde to glycine betaine. This was done using a BADH2-specific ELISA kit (Jiangsu Jingmei Biological Technology Co., Ltd., China) on the same tissue samples. Measurements were performed on samples collected from the three independent experimental repeats described in section 2.2.

### 2.4 Analysis of osmotic adjustment compounds

This study used the following methods to determine the content of proline, soluble sugar, and soluble protein in the roots and leaves of *G. uralensis*. The proline content was determined using the acid indanone method (Tirani et al., 2013) combined with the Solarbio assay kit (BC0250); The anthrone method (Aragão et al., 2015) was used in combination with the Solarbio reagent kit (BC0030) to analyze the soluble sugar content; The soluble protein (SP) content was determined by the Coomassie Brilliant Blue G-250 method (Zhou et al., 2021) using the Jiangsu Jingmei Biotechnology Co., Ltd. kit (JM-110029P2). All measurements were conducted strictly in accordance with the operating procedures outlined in the instructions of each reagent kit. Measurements were performed on samples collected from the three independent experimental repeats described in section 2.2.

### 2.5 Analysis of oxidative stress and antioxidant defense

Leaf relative electrical conductivity (REC) was measured with a conductivity meter (Bante 5, Shanghai Bante) (Dionisio-Sese and Tobita, 1998). Malondialdehyde (MDA) content in leaves was determined via thiobarbituric acid assay (Shi et al., 2014). Hydrogen peroxide (H_2_O_2_), superoxide anion (O_2_^-^) levels, and activities of superoxide dismutase (SOD), peroxidase (POD), catalase (CAT), and ascorbate peroxidase (APX) in roots and leaves were analyzed using commercial kits (Solarbio, Beijing; H_2_O_2_-BC3590, O_2_^-^-BC1290, SOD-BC0170, POD-BC0095, CAT-C017, APX-BC0220). Ascorbic acid (AsA) content was assessed by 2,6-dichlorophenol indophenol (DCPIP) method (Zhang et al., 2021). Measurements were performed on samples collected from the three independent experimental repeats described in section 2.2.

### 2.6 Observation of salt secretion behavior and ion determination

The salt glands and stomata on the abaxial surface of the leaves were observed using a scanning electron microscope (SU8010, Hitachi High-Tech, Japan), and the images were captured for documentation. Following the methodology of Newete et al (Newete et al., 2020), leaf area was scanned using an image scanner (WinRHIZO LA 2400, Epson, Japan), while K^+^, Na^+^, and Ca²^+^ concentrations were quantified by atomic absorption spectrophotometry (Agilent 240DUO, Thermo Fisher Scientific, USA). Foliar salt excretion rates were calculated using the following formula: (ng·cm^-2^·d^-1^) = Secreted ion (ng)/[leaf area (cm²) × 7 days]. Measurements were performed on samples collected from the three independent experimental repeats described in section 2.2.

### 2.7 Transcriptome sequencing and data analysis

RNA sequencing was performed on leaf samples from the four treatment groups (CK, S, S+GB+6h, and S+GB+24h) using one biological replicate (pot) from each of the three independent experimental repeats described in section 2.2 (n=3). Total RNA was extracted using the Total RNA Extractor (Trizol, Sangon Biotech, Shanghai, China), and RNA concentration and integrity were evaluated. Libraries were constructed using the NEBNext^®^ Ultra™ RNA Library Prep Kit and quantified via qRT-PCR. Libraries were pooled based on effective concentrations and the sequencing requirements for Illumina platforms. Reference genome and annotation files were obtained from the genome database (http://ngs-data-archive.psc.riken.jp/gur-genome/index.pl) (Yang et al., 2022). Clean reads were mapped to the reference genome using HISAT2 v2.0.5, and read counts per gene were calculated with Featurecounts. Data analysis included correlation analysis, Principal Component Analysis (PCA), and clustering analysis. Differentially expressed genes (DEGs) were identified using DESeq2 (v1.16.1) with thresholds of an adjusted *p* < 0.05 and |Log_2_FC| ≥ 1.5. GO enrichment analysis (Gene Ontology; http://www.geneontology.org) and KEGG pathway analysis (Kyoto Encyclopedia of Genes and Genomes; http://www.genome.jp/kegg/) were performed for DEGs.

### 2.8 RT-qPCR and analysis

The expression levels of five randomly selected DEGs were validated by RT-qPCR using Actin2 as the reference gene, with analysis performed by Shanghai Lingen Biotechnology Co., Ltd (Shanghai, China). Each gene was subjected to one biological replicate (pot) from each of the three independent experimental repeats described in section 2.2 (n=3). (**Supplementary Table 1**). The experimental procedure included the following steps: DNA contamination was eliminated from total RNA samples using the gDNA Eraser system. RNA was converted into cDNA using the PrimeScript^®^ RT Enzyme Mix I. qPCR was performed using specific primers and qPCR Mix. The relative expression levels of target genes were calculated using the 2^-ΔΔCt^ method (Kenneth and Thomas, 2001) with reference to the internal control gene *Actin2*.

### 2.9 Metabolomic profiling and data analysis

Non-targeted metabolomics analysis was performed on leaf samples from the four treatment groups (CK, S, S+GB+6h, and S+GB+24h) using one biological replicate (pot) from each of the three independent experimental repeats described in section 2.2 (n=3). Tissues (100 mg) were individually ground in liquid nitrogen. The homogenate was resuspended in prechilled 80% methanol and vortex-mixed thoroughly. After 5-min incubation on ice, samples were centrifuged at 15,000 g, 4°C for 20 min. Aliquots of supernatant were diluted with LC-MS grade water to a final concentration of 53% methanol. The samples were subsequently transferred to a fresh Eppendorf tube and then were centrifuged at 15000 g, 4°C for 20 min. Finally, the supernatant was injected into the LC-MS/MS system analysis. UHPLC-MS/MS analyses were performed using a Vanquish UHPLC system (Thermo Fisher, Germany) coupled to an Orbitrap Q Exactive™ HF mass spectrometer (Thermo Fisher, Germany) in Novogene Co., Ltd. (Beijing, China). Samples were injected onto a Hypersil Gold column (C18, 100×2.1 mm, 1.9μm) at 40°C using a 17-min linear gradient at a flow rate of 0.2 mL/min. The eluents for the positive polarity mode were eluent A (0.1% FA in Water) and eluent B (Methanol). The eluents for the negative polarity mode were eluent A (5 mM ammonium acetate, pH 9.0) and eluent B (Methanol). The solvent gradient was set as follows: 0-1.5 min, 98% A/2% B; 1.5–3 min, 15% A/85% B; 3–10 min, 0% A/100% B; 10-10.1 min, 98% A/2% B; 10.1–12 min, 98% A/2% B (total run time: 12 min). The Q Exactive™ HF mass spectrometer was operated in positive/negative polarity mode with a spray voltage of 3.5 kV, capillary temperature of 320°C, sheath gas flow rate of 35 psi and aux gas flow rate of 10 L/min, S-lens RF level of 60, Aux gas heater temperature of 350°C.

The raw data files generated by UHPLC-MS/MS were processed using the Compound Discoverer 3.1 (CD3.1, Thermo Fisher) to perform peak alignment, peak picking, and quantitation for each metabolite. The main parameters were set as follows: retention time tolerance, 0.2 minutes; actual mass tolerance, 5ppm; signal intensity tolerance, 30%; signal/noise ratio, 3; and minimum intensity. After that, peak intensities were normalized to the total spectral intensity. The normalized data were used to predict the molecular formula based on additive ions, molecular ion peaks and fragment ions. And then peaks were matched with the mzCloud (https://www.mzcloud.org/), mzVault and Mass List database to obtain the accurate qualitative and relative quantitative results. Quality control: Metabolites with >30% CV in pooled QC samples were excluded. Data from positive and negative ion modes were merged and subjected to multivariate statistical analyses in the CentOS 6.6 environment using R (v4.0.3) and Python (v3.8). These analyses included correlation analysis, orthogonal partial least squares discriminant analysis (PLS-DA), and hierarchical cluster analysis to evaluate the stability of the experimental system. Differential metabolites (DMs) were identified by combining VIP >1 (from PLS-DA), *p* < 0.05 (one-way ANOVA), and |Log_2_FC| ≥ 1 thresholds. The same criteria were applied to KEGG pathway enrichment analysis. A multi-omics joint analysis was performed by extracting the KEGG pathways that were co-enriched in both the transcriptome and metabolome. Enrichment plots were generated using ggplot2 (v3.3.5), and an interaction network between DEGs and DMs was constructed based on the Pearson correlation coefficient (|r| > 0.8, *p* < 0.05).

### 2.10 Determination of biomass

Ten seedlings of *G.uralensis* were randomly selected from each treatment. After removing surface impurities, the surface water was dried with absorbent paper. Roots, stems and leaves were separated, and oven-dried at 80°C until a constant weight. The dry weight of each organ was measured using a precision balance (BS423 S, Sartorius, Germany) with a sensitivity of 0.001 g. Measurements were performed on samples collected from the three independent experimental repeats described in section 2.2.

### 2.11 Data analysis of morphological and physiological indices

Statistical analyses were performed using SPSS 20.0 (IBM Corp., New York, USA) software. Differences among treatments were assessed by one-way ANOVA with LSD *post hoc* test (*p* < 0.05). Results were expressed as mean ± standard deviation, and graphs were created using OriginPro 2022b (Electronic Arts Inc., New York, USA).

## 3 Results

### 3.1 Exogenous GB increased the biomass of *G. uralensis* seedlings under salt stress

**Figure 1:**
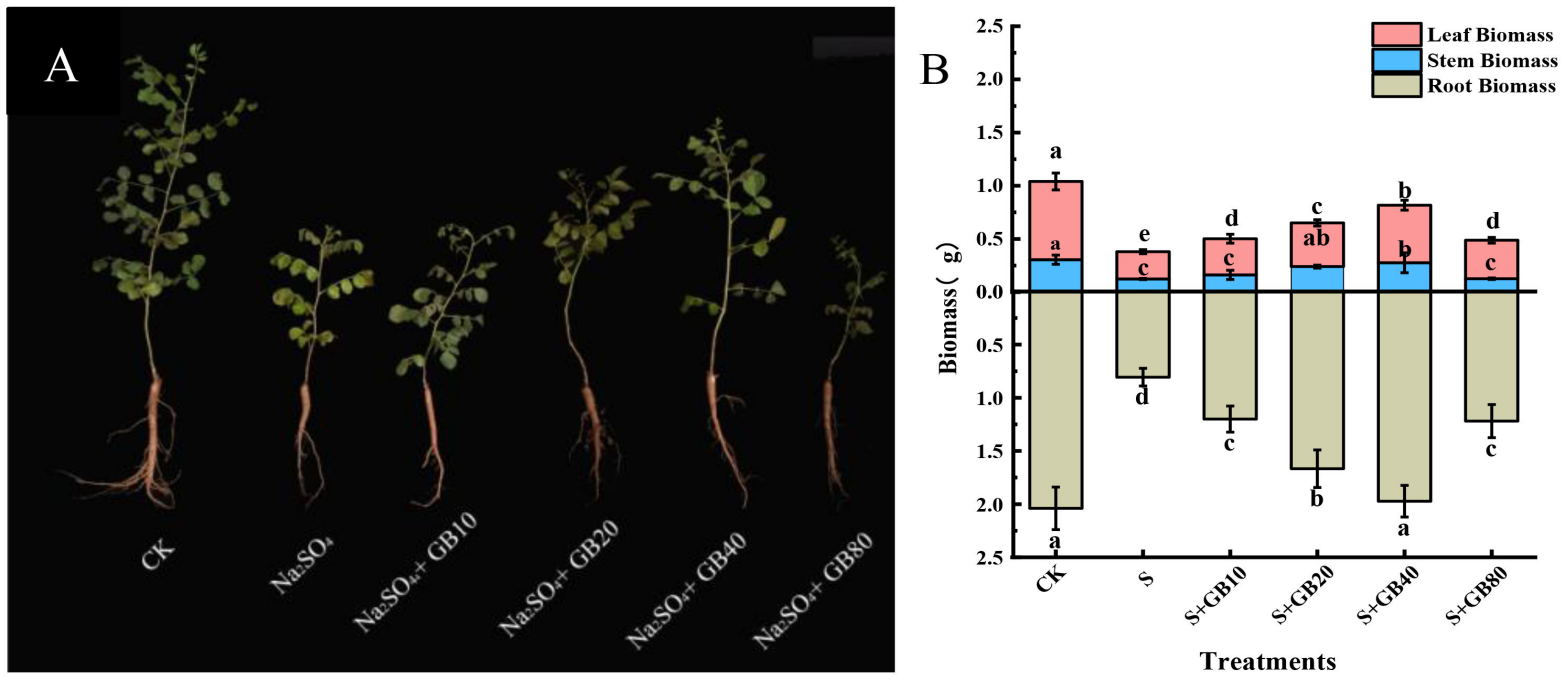
The effect of salt stress and application of GB on the growth **(A)** and biomass **(B)** of seedlings of *G.uralensis*. Data are presented as the mean ± SD (n = 30). Different lowercase letters indicate significant differences among treatments at *p* ≤ 0.05.

Na_2_SO_4_ stress significantly reduced root, stem, and leaf biomass in *G.uralensis* seedling by 60.47%, 59.58%, and 65.05% respectively, compared to the CK. However, exogenous GB application effectively reversed this decline. Compared with the salt stress group (S), GB (10, 20, and 40 mM) significantly increased root biomass by 48.88%, 106.84%, and 144.68%, stem biomass by 31.36%, 94.57%, and 123.50%, and leaf biomass by 32.18%, 60.04%, and 110.47% respectively. The most pronounced biomass enhancement across all organs occurred under 40 mM GB treatment (**Figure 1**). Furthermore, root biomass at 40 mM GB showed no significant difference relative to the control (CK).

### 3.2 Exogenous GB increased the endogenous GB content of *G. uralensis* seedlings under salt stress

**Figure 2:**
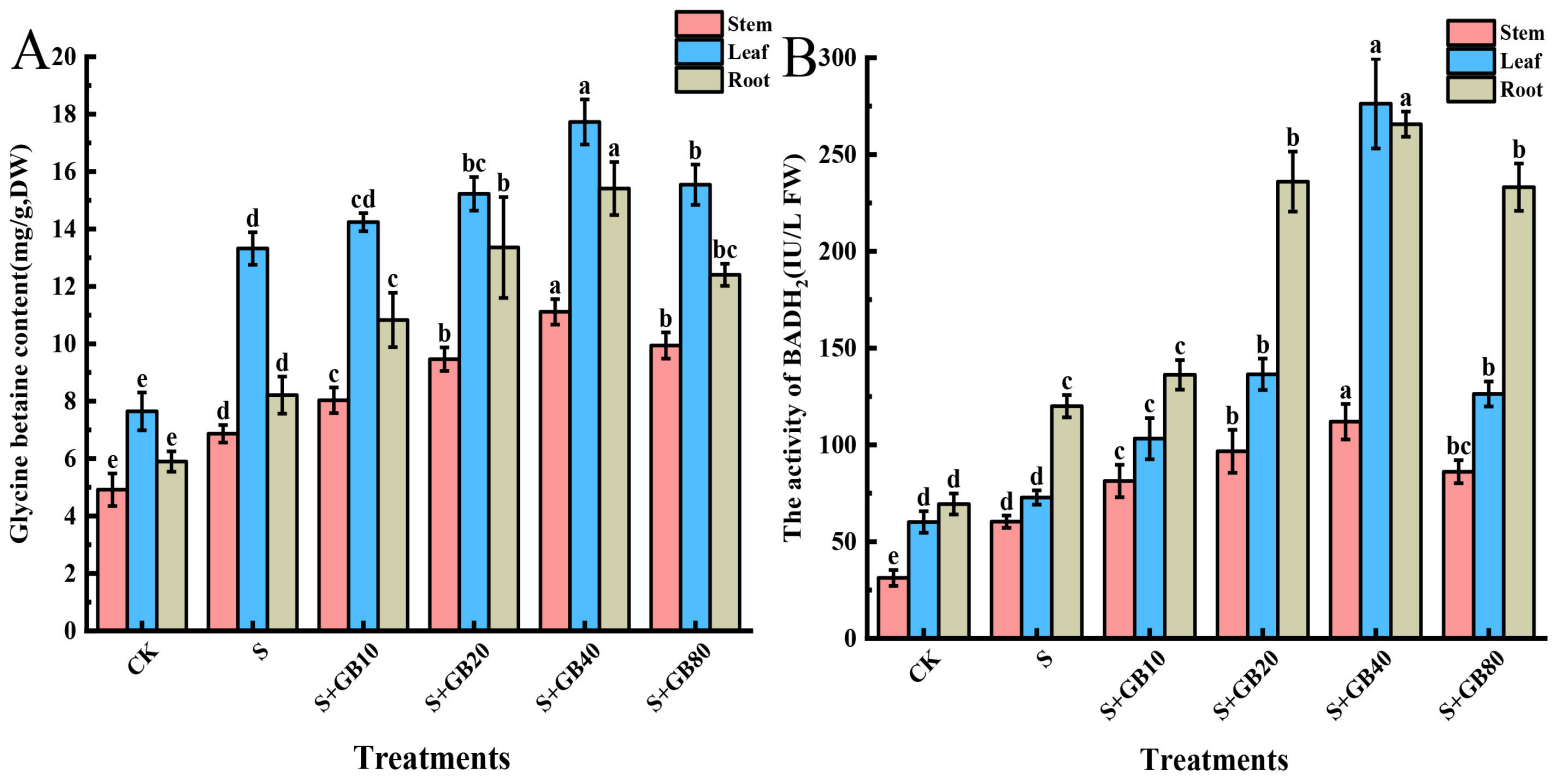
Effects of salt stress and exogenous application of GB on the endogenous GB levels **(A)** and BADH2 activity **(B)** in *G.uralensis* seedlings under salt stress. Data are presented as the mean ± SD (n = 9). Different lowercase letters indicate significant differences among treatments at *p* ≤ 0.05.

Under salt stress, the endogenous GB content increased by 39.24%, 39.65%, and 74.20%, and BADH2 activity was elevated by 72.80%, 92.98%, and 21.04% in the roots, stems, and leaves, respectively, compared to the control group (**Figures 2A, B**). The application of exogenous GB further enhanced the GB content and BADH2 activity in licorice seedlings under salt stress, leading to increases of 87.63%, 61.81%, and 33.10% in GB content in the roots, stems, and leaves, respectively; and enhancing the activity of BADH2 by 121.42%, 85.61%, and 279.59% in the same organs. Notably, endogenous GB accumulation remained highest in leaves across treatments, followed by roots and stems. Conversely, BADH2 activity consistently peaked in roots, with leaves showing intermediate levels and stems the lowest values.

### 3.3 Exogenous GB increased the content of osmotic substances in *G. uralensis* seedlings under salt stress

**Figure 3:**
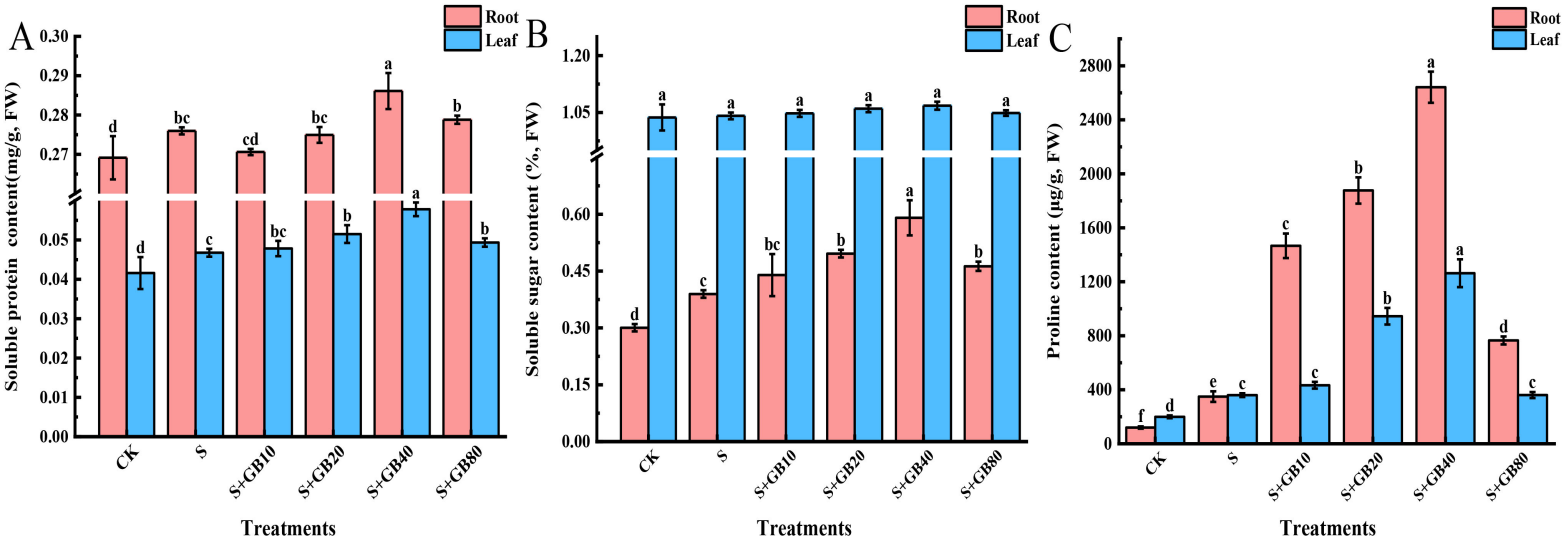
Effects of salt stress and exogenous application of GB on soluble protein **(A)**, soluble sugar **(B)** and proline **(C)** in *G.uralensis* seedlings under salt stress. Data are presented as the mean ± SD (n = 9). Different lowercase letters indicate significant differences among treatments at *p* ≤ 0.05.

Under Na_2_SO_4_ stress, the concentrations of soluble proteins, soluble sugars, and proline in seedling roots increased by 2.54%, 29.67%, and 190.86%, respectively, compared to the CK group. Similarly, soluble protein and proline levels in the leaves significantly increased by 12.42% and 80.27%, respectively. Compared to Na_2_SO_4_ treatment alone, the application of GB further enhanced the concentrations of these compounds in the roots and leaves, with maximum effects observed at 40 mM GB. At this concentration, soluble proteins, soluble sugars, and proline in the roots increased by 3.67%, 51.61%, and 655.70%, respectively, while soluble proteins and proline in the leaves increased by 23.59% and 251.09%, respectively (**Figure 3**).

### 3.4 Exogenous GB reduced the content of ROS in *G. uralensis* seedlings under salt stress

**Figure 4:**
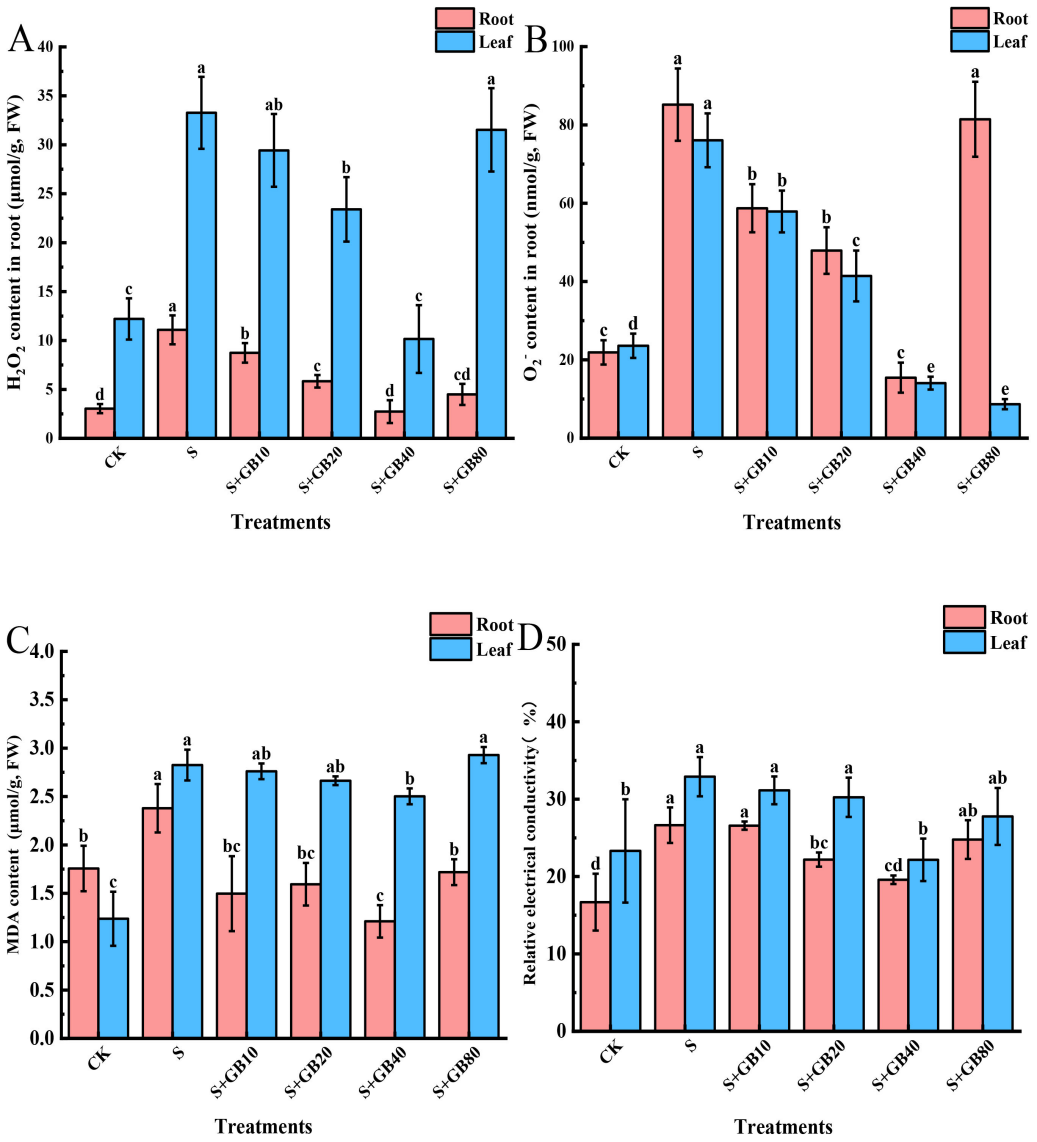
Effects of salt stress and exogenous application of GB on H_2_O_2_ **(A)**, O_2_^−^ **(B)**, MDA **(C)**, and relative electrical conductivity **(D)** in *G.uralensis* seedlings under salt stress. Data are presented as the mean ± SD (n = 9). Different lowercase letters indicate significant differences among treatments at *p* ≤ 0.05.

Na_2_SO_4_ treatment increased the concentrations of H_2_O_2_, O_2_^−^, MDA, and relative electrical conductivity in both roots and leaves (**Figure 4**). These indicators initially decreased and then increased with rising GB concentrations, showing a lowest level under 40 mM GB treatment. Compared to Na_2_SO_4_ treatment, the reductions in the roots were 75.32%, 81.86%, 96.57%, and 26.49%, respectively; in the leaves, the reductions were 69.47%, 81.54%, 12.93%, and 32.65%, respectively.

### 3.5 Exogenous GB increased the activity of antioxidant enzymes in *G. uralensis* seedlings under salt stress

**Figure 5:**
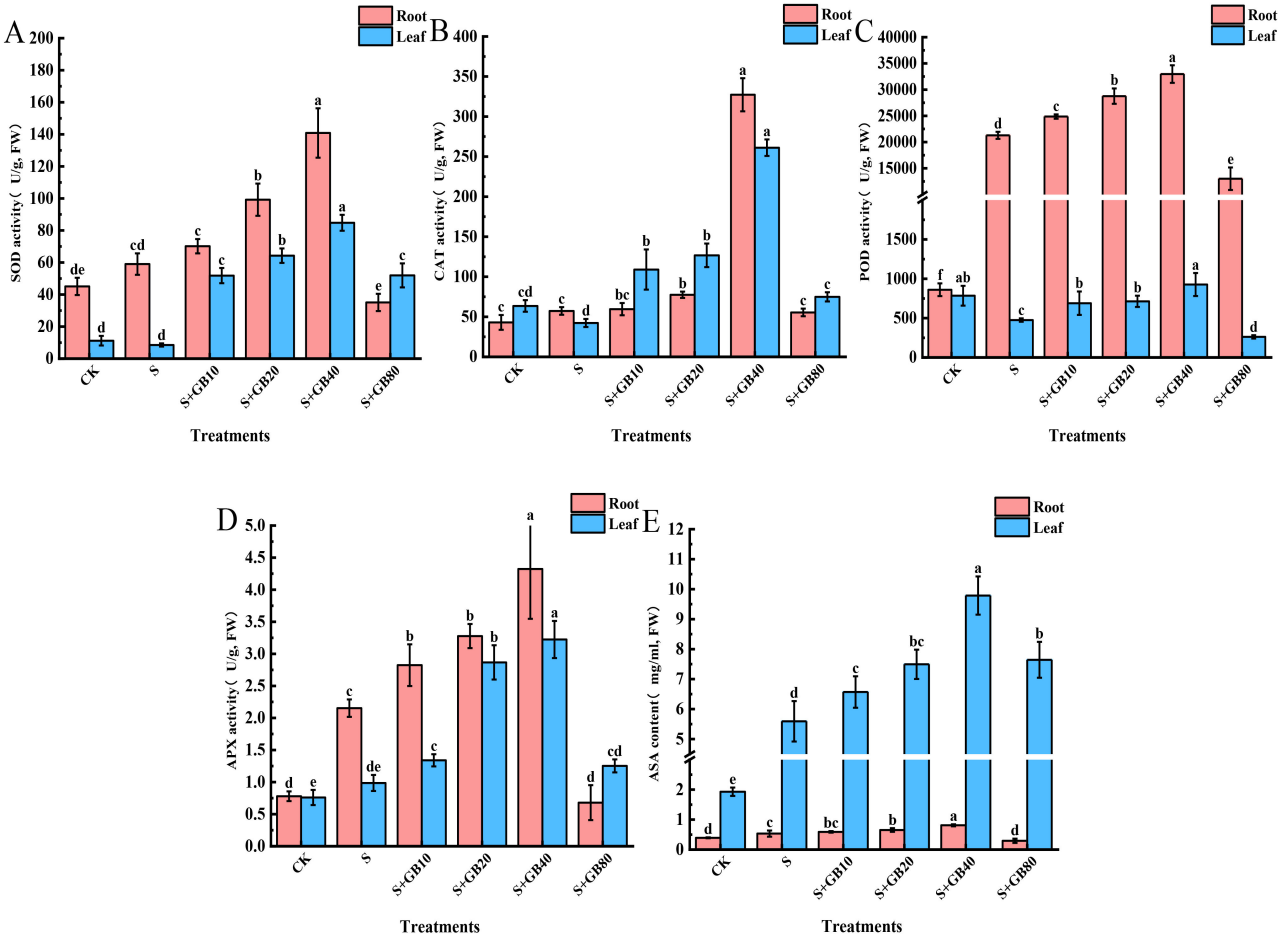
Effects of salt stress and exogenous application of GB on antioxidant enzymes SOD **(A)**, CAT **(B)**, POD **(C)**, and APX **(D)**, as well as the AsA **(E)** content in *G.uralensis* seedlings under salt stress. Data are presented as the mean ± SD (n = 9). Different lowercase letters indicate significant differences among treatments at *p* ≤ 0.05.

Compared to the control group, Na_2_SO_4_ significantly increased the activities of antioxidant enzymes SOD (**Figure 5A**), CAT (**Figure 5B**), POD (**Figure 5C**), and APX (**Figure 5D**), as well as the AsA content (**Figure 5E**) in seedling roots, while decreasing the activities of SOD, CAT, POD, and APX in leaves but increasing AsA concentration. Following exogenous GB treatment, the activities of SOD, CAT, POD, APX, and AsA content in both roots and leaves showed a pattern of initial increase followed by a decrease as GB concentration rose, reaching their peaks under 40 mM GB treatment. Compared to Na_2_SO_4_ treatment, these indicators increased by 138.58%, 471.15%, 54.93%, 100.77%, and 51.88% in the roots, and by 893.71%, 518.12%, 50.00%, 226.69%, and 74.97% in leaves, respectively.

### 3.6 Exogenous GB reduced the salt secretion rate of *G. uralensis* seedlings under saline stress

**Figure 6:**
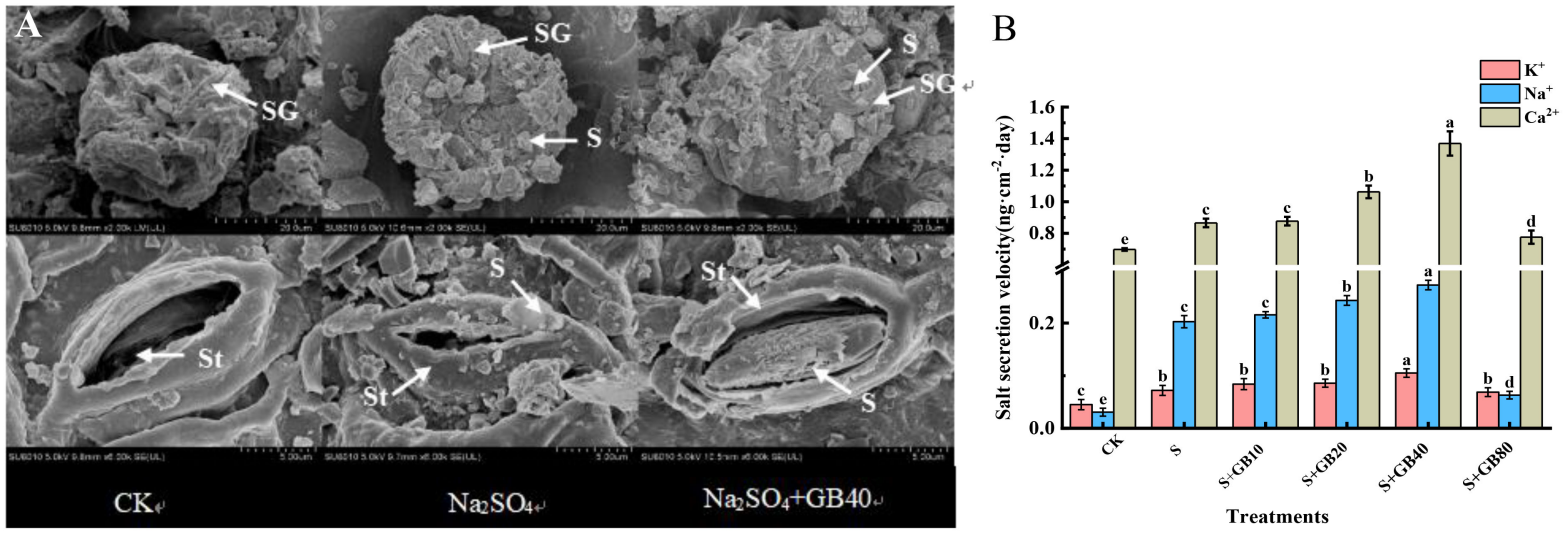
The effects of salt stress and exogenous GB on salt secretion **(A)** and K^+^, Na^+^, and Ca^2+^ secretion rate **(B)** of *G*. *uralensis.* Note: SG, salt gland; St, stoma; S, salt. Data are presented as the mean ± SD (n = 9). Different lowercase letters indicate significant differences among treatments at *p* ≤ 0.05.

The stomata and salt glands on leaves of *G. uralensis* function as salt-secreting structures. Control sample analysis revealed Ca²^+^ as the predominant ion in exudates. Under both sole Na_2_SO^4^ and Na_2_SO^4^+GB treatments, significant salt accumulation was observed surrounding these structures (**Figure 6A**). Compared to the control, Na_2_SO_4_ treatment significantly increased the secretion rates of K^+^, Na^+^, and Ca^2+^ in the leaves by 60.00%, 560.87%, and 24.08%, respectively (**Figure 6B**). With increasing GB concentrations, the secretion rates of K^+^, Na^+^, and Ca^2+^ in seedlings were further enhanced. At a concentration of 40 mM GB, the secretion rates reached their maximum values, increasing by 145.83%, 134.38%, and 56.14%, respectively, compared to treatment with Na_2_SO_4_ alone.

### 3.7 Transcriptome results

Transcriptomic analysis was performed on *G. uralensis* leaf samples collected at 6 h and 24 h after GB treatment, resulting in the construction of 12 cDNA libraries. High-throughput sequencing yielded 506,300,074 clean reads across the 12 samples, representing 97% of the total raw reads. The Q20 and Q30 base percentages exceeded 97% and 93.02%, respectively. The GC content ranged from 44.51% to 45.43% (**Supplementary Table 2**).

**Figure 7:**
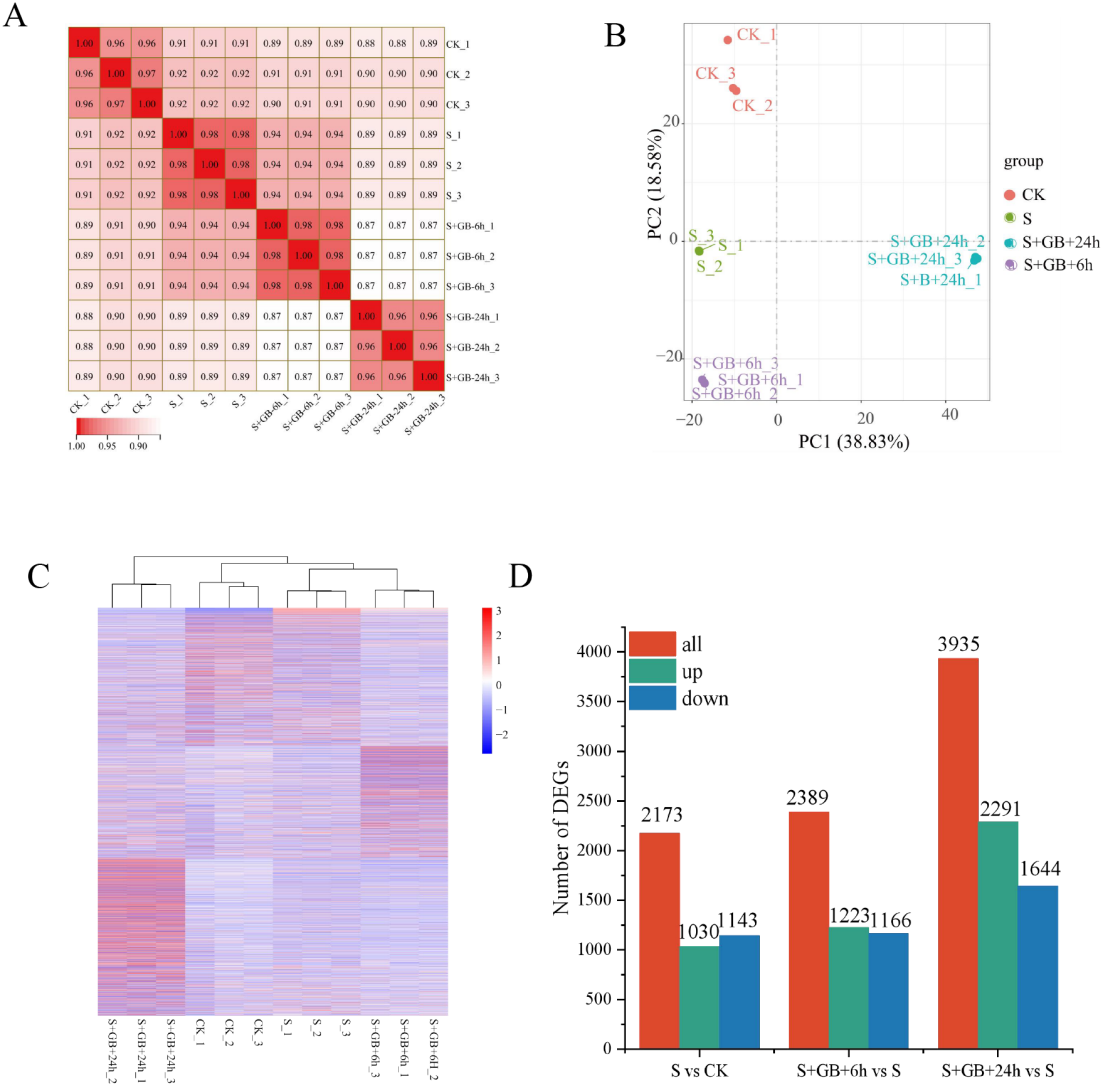
Comprehensive analysis of transcriptome data and identification of differentially expressed genes (DEGs) in *G*. *uralensis* under different treatments. **(A)** Heatmap of Pearson correlation coefficients (r values; values closer to 1 indicate higher reproducibility among biological replicates). **(B)** Principal component analysis (PCA) plot (Points represent samples colored by experimental group; PC1 (horizontal axis) and PC2 (vertical axis) denote the first and second principal components). **(C)** Hierarchical clustering heatmap (Branch lengths on the vertical axis reflect sample similarity). **(D)** Number of DEGs. (CK: Control group; S: Na_2_SO_4_ stress group; S+GB+6h: Na_2_SO_4_ stress + Glycine betaine treatment for 6 h group; S+GB+24h: Na_2_SO_4_ stress + Glycine betaine treatment for 24 h group.).

The correlation index R² among samples within each treatment group exceeded 0.96, indicating high reproducibility within group (**Figure 7A**). PCA analysis showed that the distances between samples are relatively close, suggesting minimal differences among samples within the group; however, the treatments are significantly separated along PCA1 and PCA2, reflecting significant inter-group differences. This suggests that Na_2_SO_4_ treatment and the combined treatment of Na_2_SO_4_ and GB caused substantial changes in *G. uralensis* gene expression (**Figure 7B**). The treatment group S+GB+24h is distinctly separated from other groups, highlighting its markedly different gene expression profile (**Figure 7C**). Gene expression differences among samples were calculated based on FPKM. Compared to the CK group, the salt treatment group (S) identified DEGs, including 1030 upregulated and 1143 downregulated. Compared to the S group, the S+GB+6h group identified DEGs, including 1223 upregulated and 1166 downregulated; compared to the S group, the S+GB+24h group identified DEGs, including 2291 upregulated and 1644 downregulated (**Figure 7D**).

We classified the DEGs into three categories based on GO terms to elucidate their functions: biological processes, cellular components, and molecular functions.

**Figure 8:**
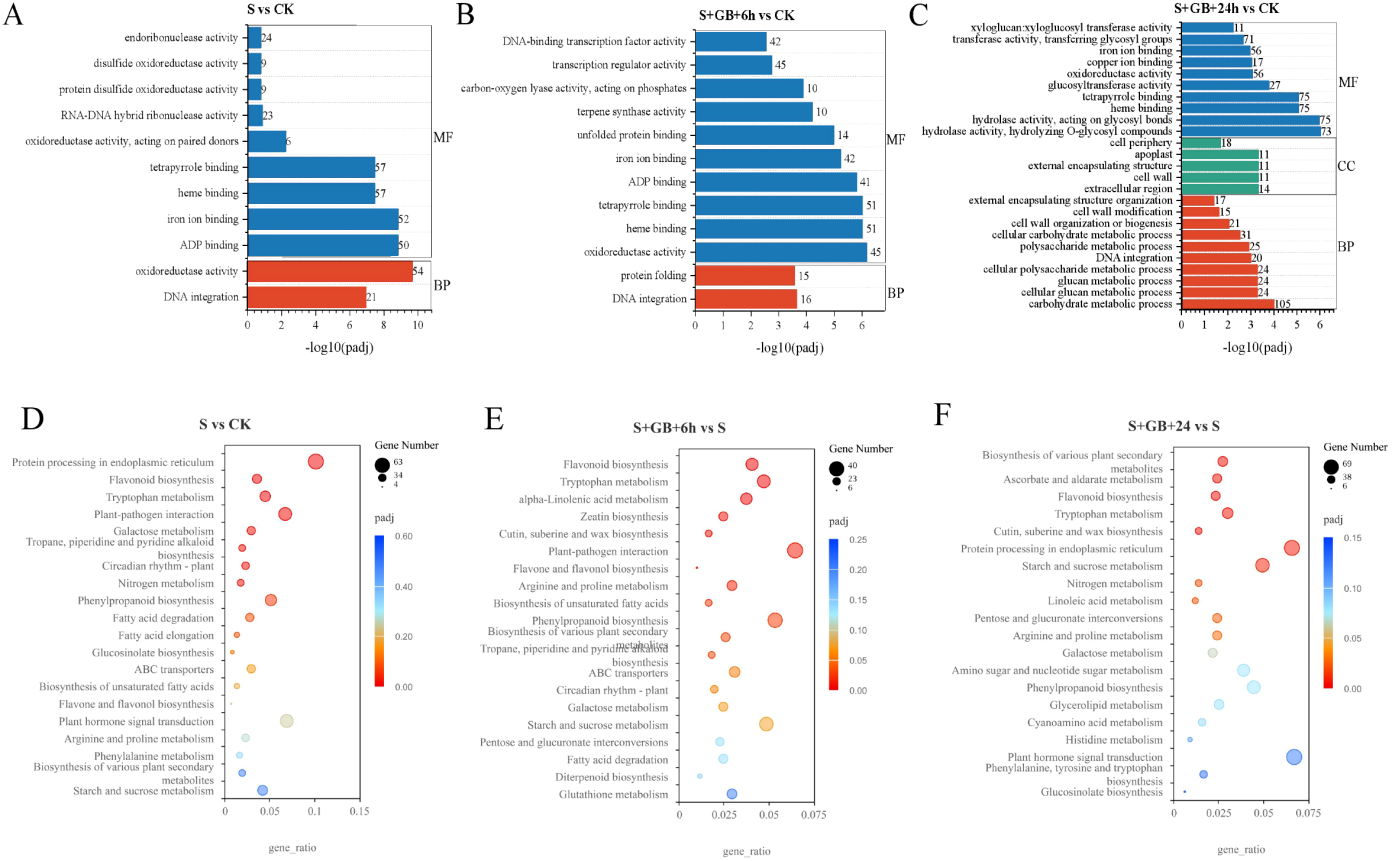
GO and KEGG enrichment analysis of differentially expressed genes (DEGs) from S vs CK **(A)**, S+GB+6h vs S **(B)**, and S+GB+24h vs S **(C)**. **(A-C)** GO enrichment analysis of DEGs. Bars represent significantly enriched terms (*P*-adj < 0.05); y-axis: GO terms grouped by ontology categories (biological process, molecular function, cellular component); x-axis: -log_10_(*P*-adj) enrichment score. **(D-F)** KEGG pathway enrichment analysis of DEGs. Bubble plots show enriched pathways; x-axis: gene ratio (number of DEGs in pathway/total DEGs); y-axis: pathway names; bubble size: number of DEGs; color gradient: enrichment significance (*P*-adj < 0.05), red = most significant). (CK: Control group; S: Na_2_SO_4_ stress group; S+GB+6h: Na_2_SO_4_ stress + Glycine betaine treatment for 6 h group; S+GB+24h: Na_2_SO_4_ stress + Glycine betaine treatment for 24 h group.).

Compared to the CK group, 496, 156, and 768 DEGs in the S group were annotated under the biological process, cellular component, and molecular function categories, respectively. In the biological process category, enriched terms included DNA integration and protein folding. In the molecular function category, enriched terms included heme binding, porphyrin binding, ADP binding, iron ion binding, unfolded protein binding, terpenoid synthase activity, carbon-oxygen lyase activity, phosphatase activity, transcription regulator activity, and DNA-binding transcription factor activity (**Figure 8A**).

Compared to the S group, 563, 121, and 848 DEGs in the S+GB+6h group were annotated under the biological process, cellular component, and molecular function categories, respectively. In the biological processes category, DNA integration was significantly enriched. In the molecular function category, enriched terms included ‘oxidoreductase activity, acting on paired donors, with incorporation or reduction of molecular oxygen’, ‘ADP binding, iron ion binding’, ‘heme binding, tetrapyrrole binding, and ‘oxidoreductase activity, acting on paired donors, with oxidation of a pair of donors resulting in the reduction of molecular oxygen to two molecules of water’ (**Figure 8B**).

Compared to the S group, 925, 254, and 1468 DEGs in the S+GB+24h group were annotated under the biological process, cellular component, and molecular function categories, respectively. In the biological process category, enriched terms included carbohydrate metabolic process, cellular glucan metabolic process, glucan metabolic process, cellular polysaccharide metabolic process, DNA integration, polysaccharide metabolic process, cellular carbohydrate metabolic process, cell wall organization or biogenesis, cell wall modification, and external encapsulating structure organization. In the cellular component category, enriched terms included extracellular region, cell wall, external encapsulating structure, exosome, and cell periphery. In the molecular function category, enriched terms included ‘hydrolase activity, hydrolyzing O-glycosyl compounds’, heme binding, tetrapyrrole binding, glucosyltransferase activity, ‘oxidoreductase activity, acting on paired donors, with incorporation or reduction of molecular oxygen’, iron ion binding, ‘transferase activity, transferring glycosyl groups’ and ‘xyloglucan: xyloglucosyl transferase activity’ (**Figure 8C**).

To gain a comprehensive understanding of the key metabolic pathways of *G. uralensis* in response to GB under salt stress, KEGG pathway enrichment analysis was conducted based on the expression profiles.

Compared to the CK group, DEGs in the S group were significantly enriched in pathways such as protein processing in the endoplasmic reticulum, flavonoid biosynthesis, tryptophan metabolism, plant-pathogen interactions, galactose metabolism, terpene biosynthesis, piperidine and pyridine alkaloid biosynthesis, circadian rhythm in plants, and nitrogen metabolism. While the number of upregulated genes in the protein processing pathway of the endoplasmic reticulum exceeded that of downregulated ones, other pathways exhibited fewer upregulated genes than downregulated ones, suggesting that salt stress significantly inhibits gene expression in most metabolic pathways (**Figure 8D**).

Compared to the S group, DEGs in the S+GB+6h group were primarily enriched in pathways such as flavonoid biosynthesis, tryptophan metabolism, α-linolenic acid metabolism, zeatin biosynthesis, cutin, suberin and wax biosynthesis, plant-pathogen interactions, flavonoid and flavonol biosynthesis, arginine and proline metabolism, unsaturated fatty acid biosynthesis, phenylpropanoid biosynthesis, various secondary metabolite biosynthesis, and terpene, piperidine, and pyridine alkaloid biosynthesis. Among these pathways, only in the zein biosynthesis and plant-pathogen interaction pathways do upregulated genes significantly outnumber downregulated ones (**Figure 8E**).

Compared to the S group, DEGs in the S+GB+24h group were significantly enriched in pathways associated with the biosynthesis of plant secondary metabolites, ascorbate and aldaric acid metabolism, flavonoid biosynthesis, tryptophan metabolism, cutin, suberin, and wax biosynthesis, protein processing in the endoplasmic reticulum, starch and sucrose metabolism, nitrogen metabolism, linoleic acid metabolism, pentose and glucuronic acid interconversion, as well as arginine and proline metabolism (**Figure 8F**). Among these pathways, upregulated genes outnumbered downregulated ones in pentose and glucuronic acid interconversion, phenylpropanoid biosynthesis, cutin, suberin, and wax biosynthesis, flavonoid biosynthesis, starch and sucrose metabolism, linoleic acid metabolism, tryptophan metabolism, fatty acid biosynthesis, phenylalanine, tyrosine, and tryptophan biosynthesis, cyanamide metabolism, and brassinolide biosynthesis (**Supplementary Table 3**).

To validate the RNA-seq data, five genes were randomly selected for QRT-PCR analysis, including Glyur001213s00027166, Glyur000019s00002088, Glyur000815s00035014, Glyur000178s00013231, and Glyur000314s00018014, to assess their expression patterns. The expression patterns of these selected genes were further confirmed by qPCR analysis, which were highly consistent with the RNA-seq data (**Supplementary Figure 1**). Notably, the validated genes exhibited distinct temporal expression patterns, suggestive of early versus late functional roles in the GB response. While a minor quantification discrepancy was observed for one gene at a single time point—potentially due to technical factors like alternative splicing—the overwhelming concordance between RNA-seq and qPCR data confirms the robustness of our transcriptomic analysis.

### 3.8 Metabolomics results

**Figure 9:**
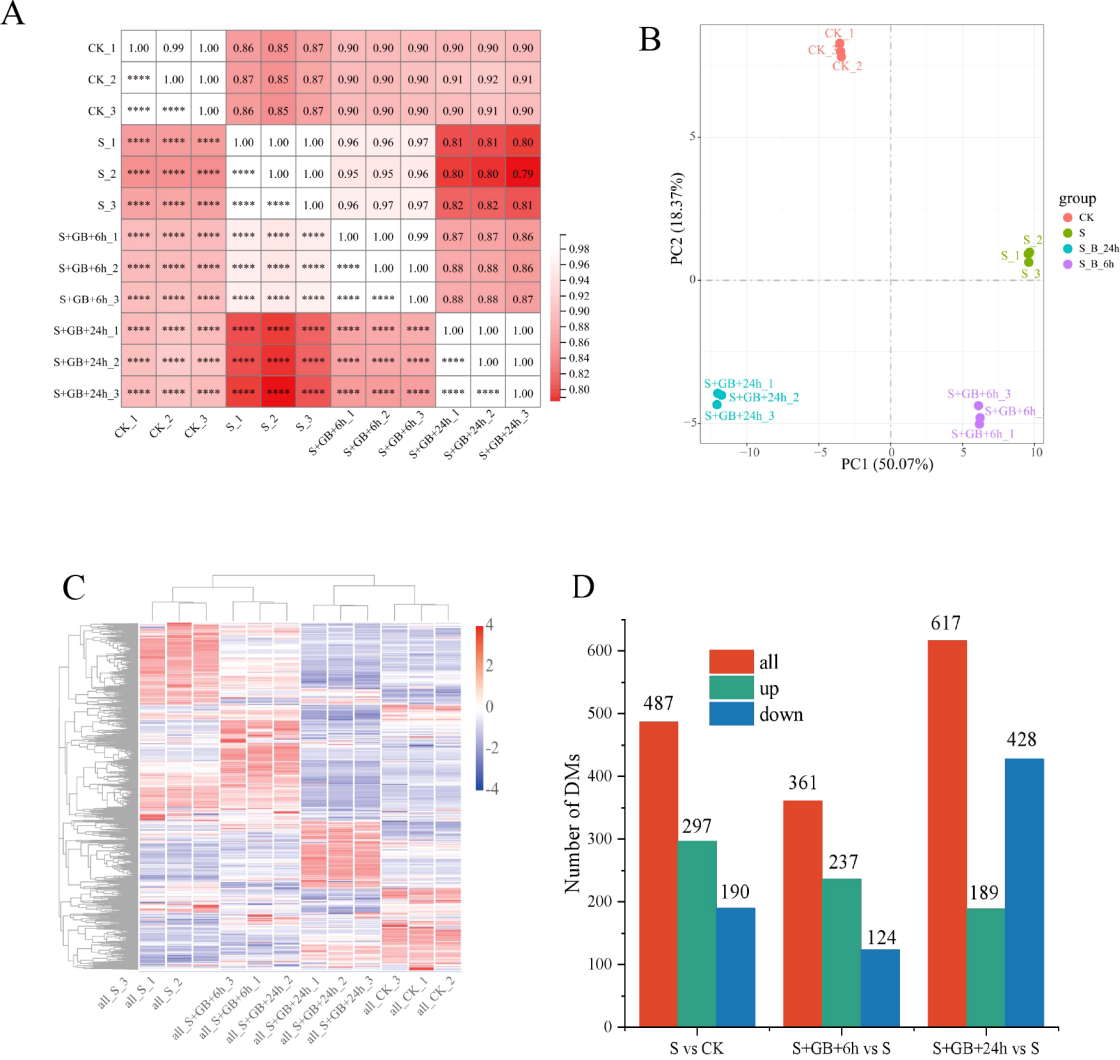
Comprehensive analysis of metabolome data and identification of differentially expressed metabolites (DMs) in *G*. *uralensis* under different treatments. **(A)** Sample correlation heatmap (R values; darker red indicates stronger inter-sample correlation). **(B)**Principal component analysis (PCA) plot. Points represent samples colored by experimental group; PC1 (x-axis) and PC2 (y-axis) denote primary variance components. **(C)** Hierarchical clustering heatmap of samples. Dendrogram branch lengths reflect sample similarity. **(D)** Number of DMs in samples. (CK: Control group; S: Na_2_SO_4_ stress group; S+GB+6h: Na_2_SO_4_ stress + Glycine betaine treatment for 6 h group; S+GB+24h: Na_2_SO_4_ stress + Glycine betaine treatment for 24 h group.).

The correlation coefficient (R²) among samples in the same treatment group was above 0.8, demonstrating high reproducibility (**Figure 9A**). The distinct separation of groups in PCA1 and PCA2 indicates significant differences among the treatment groups (**Figure 9B**). Treatment with Na_2_SO^4^ alone and in combination with GB caused significant changes in metabolite content. Samples S and S+GB+6h clustered together, reflecting similar metabolite compositions and contents, while CK and S+GB+24h also clustered together, suggesting relatively close metabolite profiles (**Figure 9C**). Compared to the CK group, the S group contained 487 DMs, with 297 upregulated and 190 downregulated. Compared to the S group, the S+GB+6h group contained 361 DMs, with 237 upregulated and 124 downregulated. Similarly, compared to the S group, the S+GB+24h group contained 617 DMs, with 189 upregulated and 428 downregulated (**Figure 9D**).

**Figure 10:**
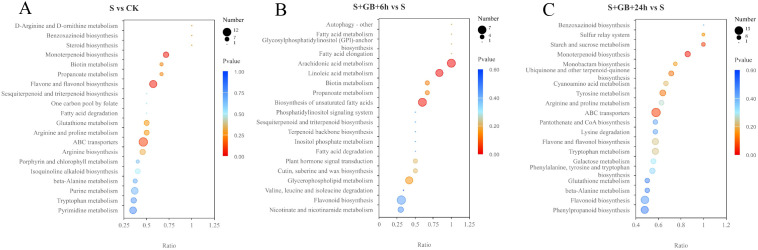
KEGG enrichment analysis of differentially expressed metabolites (DMs) from S vs CK **(A)**, S+GB+6h vs S **(B)**, and S+GB+24h vs S **(C)**. (CK: Control group; S: Na_2_SO_4_ stress group; S+GB+6h: Na_2_SO_4_ stress + Glycine betaine treatment for 6 h group; S+GB+24h: Na_2_SO_4_ stress + Glycine betaine treatment for 24 h group.) Bubble plot showing significantly enriched pathways; Horizontal axis: Rich factor (number of DMs in pathway/total DMs); Vertical axis: Pathway names; Bubble size: Number of metabolites annotated; Color gradient: -log_10_(*P*-value) (red = highest significance, blue = lowest).

**Figure 11:**
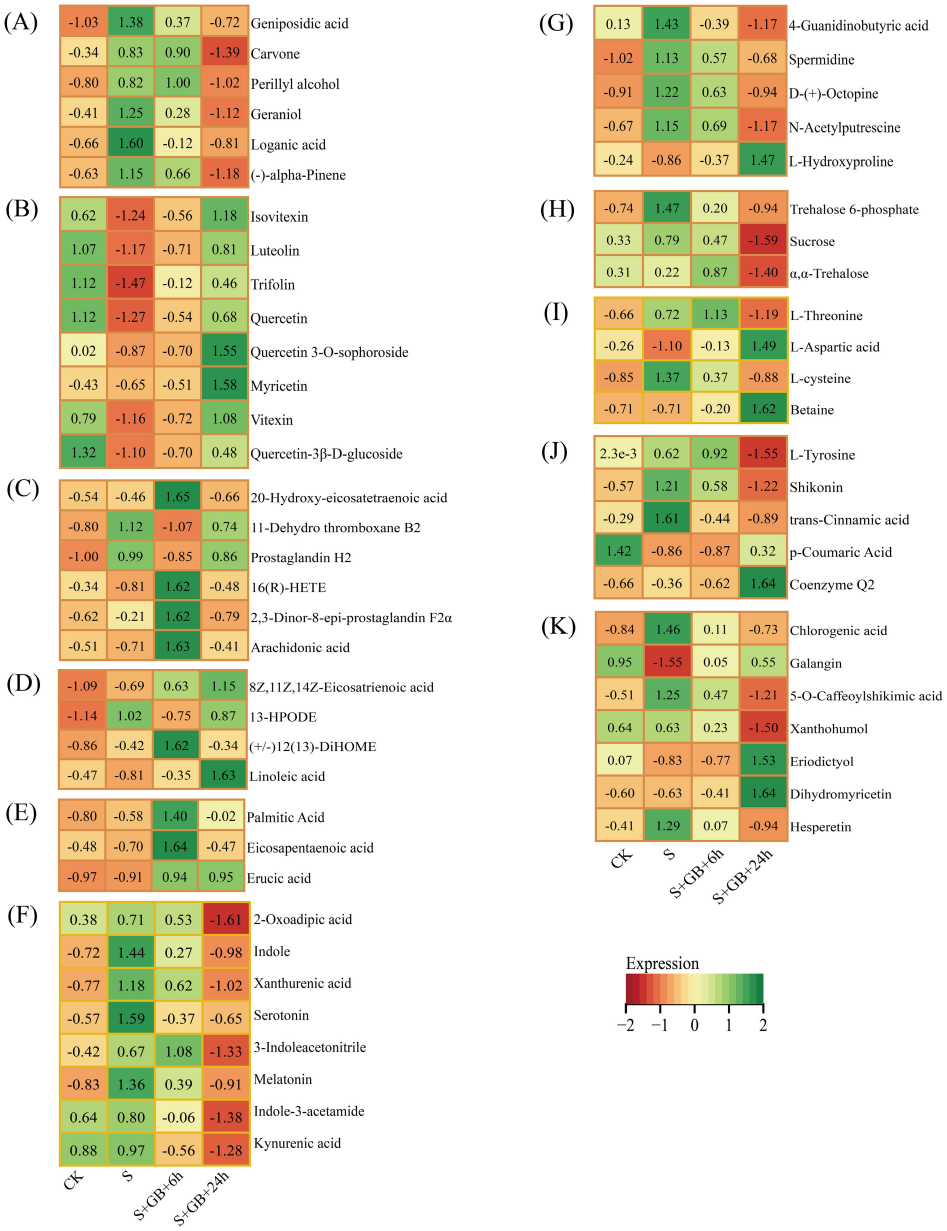
Dynamic changes in metabolites related to significantly enriched DMs. **(A)** Monoterpenoid biosynthesis; **(B)** Flavone and flavonol biosynthesis; **(C)** Arachidonic acid metabolism; **(D)** Linoleic acid metabolism; **(E)** Biosynthesis of unsaturated fatty acids; **(F)** Tryptophan metabolism; **(G)** Arginine and proline metabolism; **(H)** Starch and sucrose metabolism; **(I)** Glycine, serine, and threonine metabolism; **(J)** Ubiquinone and other terpenoid-quinone biosynthesis; **(K)** Flavonoid biosynthesis. (CK: Control group; S: Na₂SO₄ stress group; S+GB+6h: Na₂SO₄ stress + Glycine betaine treatment for 6 h group; S+GB+6h: Na₂SO₄ stress + Glycine betaine treatment for 24 h group.)

The KEGG enrichment analysis of DMs revealed that, compared to the CK group, the S group enriched 108 DMs mapped to 30 metabolic pathways. Among these, the monoterpenoid biosynthesis pathway and the flavone and flavonol biosynthesis pathways were significantly enriched (**Figure 10A**). Compared to the S group, the S+GB+6h group enriched 79 DMs mapped to 35 metabolic pathways. Notably, pathways such as arachidonic acid metabolism, linoleic acid metabolism, and unsaturated fatty acid biosynthesis were significantly enriched (**Figure 10B**). Compared with the S group, the S+GB+24h group enriched 138 DMs mapped to 36 metabolic pathways. The monoterpenoid biosynthesis pathway was significantly enriched (**Figure 10C**). In the monoterpenoid biosynthesis pathway, the content of most compounds significantly increased (**Figure 11D**), while in the flavone and flavonol biosynthesis pathways, the content of compounds significantly decreased (**Figure 11F**). Compared to the S group, the S+GB+6h group enriched 79 DMs mapped to 35 metabolic pathways. Notably, pathways such as arachidonic acid metabolism, linoleic acid metabolism, and unsaturated fatty acid biosynthesis were significantly enriched (**Figure 10B**). The content of most lipid compounds in these pathways significantly increased after 6 h of GB treatment (**Figures 11A–C**). Compared with the S group, the S+GB+24h group enriched 138 DMs mapped to 36 metabolic pathways. The monoterpenoid biosynthesis pathway was significantly enriched (**Figure 10C**), with the content of most compounds in this pathway significantly decreased (**Figure 11D**).

**Figure 12:**
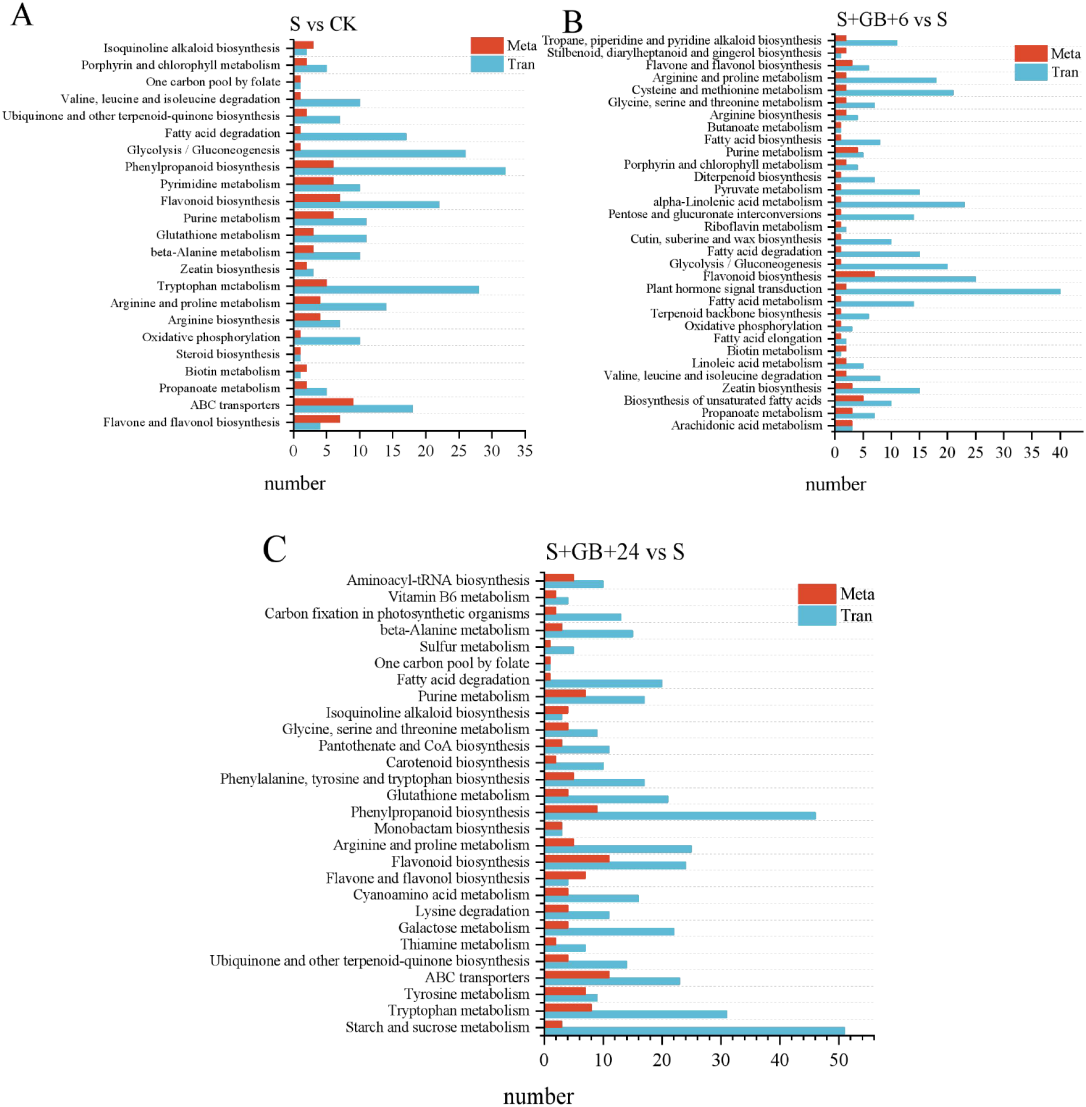
Integrated KEGG pathway enrichment analysis of differentially expressed genes (DEGs) and differential metabolites (DMs) from three comparisons: S vs CK **(A)**, S+GB+6h vs S **(B)**, and S+GB+24h vs S **(C)**. X-axis:Number of enriched metabolites/genes in the pathway. Y-axis: KEGG pathways co-enriched in metabolomics and transcriptomics. (CK: Control group; S: Na_2_SO_4_ stress group; S+GB+6h: Na_2_SO_4_ stress + Glycine betaine treatment for 6 h group; S+GB+24h: Na_2_SO_4_ stress + Glycine betaine treatment for 24 h group).

In the ubiquinone and other terpenoid-quinone biosynthesis pathway, the content of the antioxidant Coenzyme Q2 accumulated significantly after the addition of GB24h (**Figure 11E**). We also screened other metabolic pathways related to plant salt tolerance. Compared to the CK group, salt stress significantly increased the content of most compounds in the monoterpenoid biosynthesis pathway, while compounds in the flavonoid and flavonol biosynthesis pathways showed a significant decrease. After 24 h of GB treatment under salt stress, the content of most compounds in the monoterpenoid pathway decreased (**Figure 11A**), whereas compound levels in the flavonoid/flavonol biosynthesis pathway increased (**Figure 12B**). Furthermore, after 6 h of GB treatment, the majority of lipid-related compounds showed a significant increase (**Figure 11C–E**). After 24 h of GB treatment, most compounds in the tryptophan metabolism pathway decreased (**Figure 11F**), while hydroxyproline content in the arginine and proline metabolism pathways rose significantly (**Figure 11G**). Levels of trehalose-6-phosphate, sucrose α, and α-trehalose declined notably in the starch and sucrose metabolism pathways (**Figure 11H**), whereas the glycine, serine, and threonine metabolism pathways showed a significant increase in the content of L-aspartic acid and endogenous GB (**Figure 12I**). Additionally, marked accumulation of Coenzyme Q2 was observed in the ubiquinone and other terpenoid-quinone biosynthesis pathway (**Figure 11J**), and both eriodictyol and dihydromyricetin exhibited significant accumulation in the flavonoid biosynthesis pathway (**Figure 11K**).

### 3.9 Integrated analysis of transcriptomics and metabolomics

The integrated analysis of transcriptomics and metabolomics indicate that, compared to the CK group, DEGs and DMs in the S group were enriched in 23 pathways, including flavonoid and flavonol biosynthesis, ABC transporters, propanoate metabolism, biotin metabolism, steroid biosynthesis, and oxidative phosphorylation (**Figure 12A**). Compared to the S group, the S+GB+6h and S+GB+24h groups exhibited DEGs and DMs were enriched in 32 and 28 metabolic pathways, respectively. The gene expression and metabolite accumulation at these two time points displayed distinct enrichment patterns: the S+GB+6h group was primarily enriched in lipid synthesis pathways and also involved amino acid metabolism (**Figure 12B**). while the S+GB+24h group was mainly enriched in pathways related to secondary metabolite synthesis, along with carbohydrate, amino acid, and vitamin metabolism (**Figure 12C**). Furthermore, compared to the S group, the S+GB+24h group showed a significant upregulation of most DEGs in the phenylpropanoid biosynthesis and flavonoid biosynthesis pathways, while the levels of most DMs in the flavone and flavonol biosynthesis sub-pathway significantly increased. These three pathways share common metabolic routes, detailed changes in DEGs and DMs were further analyzed.

**Figure 13:**
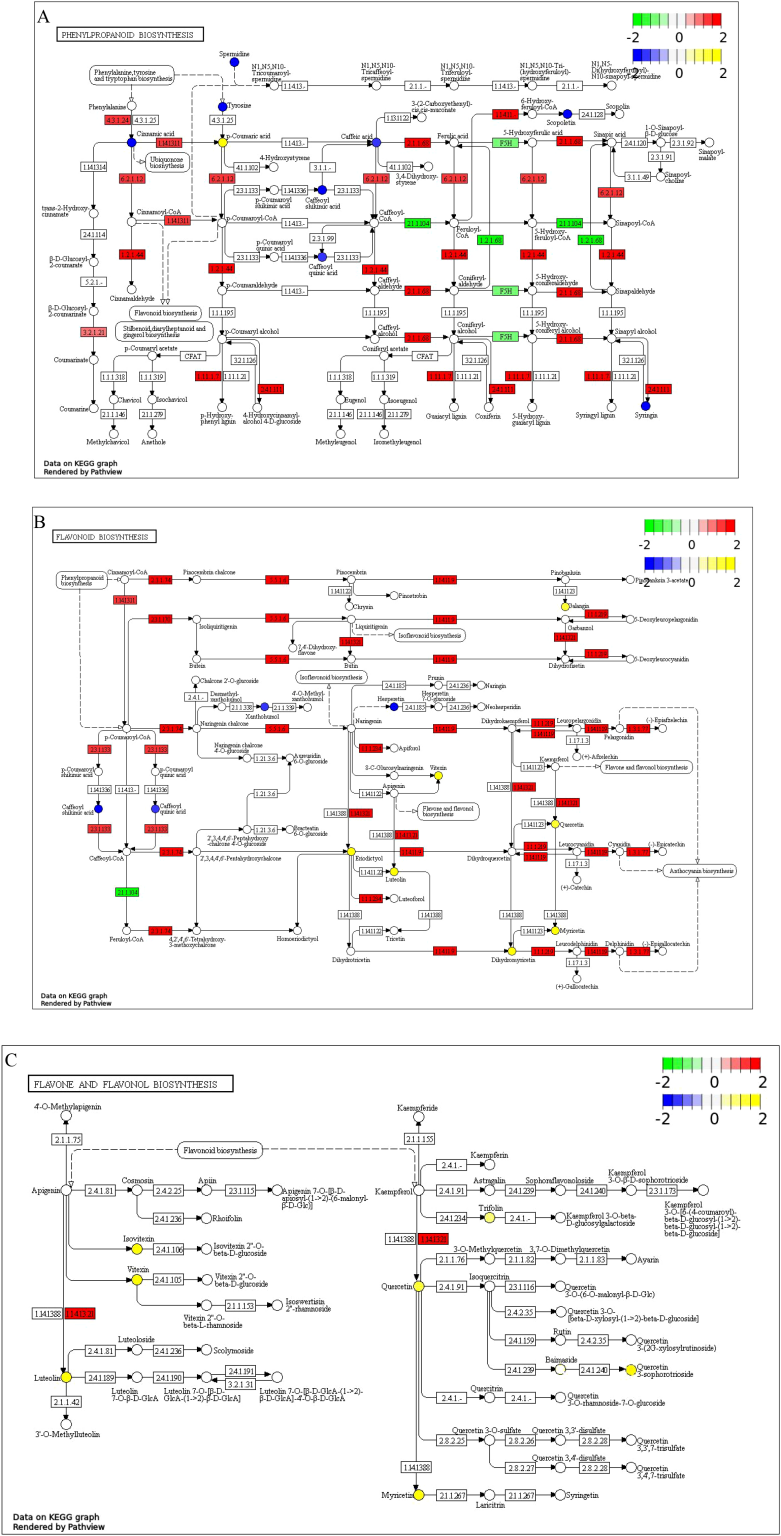
KEGG enrichment pathway map of DMs and DEGs **(A)** Phenylpropanoid biosynthesis pathway; **(B)** Flavonoid biosynthesis pathway; **(C)** Flavone and flavonol biosynthesis pathway; The log_2_(FC) values of metabolites and genes are displayed as labels; Circles represent metabolites: blue indicates downregulated DMs, and yellow indicates upregulated ones; Rectangles represent genes: green indicates DEGs with decreased expression levels, and red indicates those with increased expression levels.

The phenylpropanoid biosynthesis pathway includes 9 enriched DMs and 46 DEGs. The DMs are chlorogenic acid, L-tyrosine, 5-O-caffeoyl-mallate, spermidine, eleutheroside B, trans-cinnamic acid, p-coumaric acid, caffeic acid, and dehydroevodiamine. Except for the significant increase in p-coumaric acid content, the levels of other metabolites have significantly decreased. The DEGs are primarily annotated to enzymes including: 4-coumarate-CoA ligase (6.2.1.12), cinnamate-CoA reductase (1.2.1.44), acetylserotonin O-methyltransferase (2.1.1.68), taxifolin glucosyltransferase (2.4.1.111), and peroxidase (1.11.1.7). The expression of most genes in this pathway are significantly upregulated, including 19 genes encoding peroxidases (**Figure 13A**).

The flavonoid biosynthesis pathways has enriched 11 DMs and 24 DEGs. The DMs are chlorogenic acid, 5-O-caffeoyl-2-malonylmalic acid, flavanone, and hesperidin, whose levels significantly decreased, while those of luteolin, galangin, quercetin, myricetin, rosmarinic acid, dihydromyricetin, and vitexin significantly increased. The DEGs are primarily annotated to enzymes including: chalcone synthase (2.3.1.74), chalcone isomerase (5.5.1.6), hesperetin 3-dioxygenase (1.14.11.9), flavanone 4-reductase (1.1.1.219), anthocyanidin reductase (1.3.1.77), malonyl-CoA O-hydroxycinnamoyltransferase (2.3.1.133), flavonoid synthase (1.14.20.4), and flavonoid 3’-monooxygenase (1.14.14.82). Most of the genes encoding these enzymes were upregulated (**Figure 13B**).

The flavone and flavonol biosynthesis pathway was enriched with 6 DMs and 4 DEGs. The DMs are isovitexin, luteolin, trifolin, quercetin, quercetin 3-O-robinobioside, myricetin, and vitexin, all of which increased significantly in levels. The DEGs were annotated as flavonoid 3’-monooxygenase (1.14.13.21) and were significantly upregulated (**Figure 13C**).

To explore the relationship between DMs and DEGs in relevant pathways, Pearson’s statistical method was used to calculate the correlation coefficient (ρ) and *P*-value between the relative abundance of each DEGs and various DMs. In the phenylpropanoid biosynthesis pathway, most DEGs showed a significant positive correlation with the accumulation of p-coumaric acid (**Supplementary Figure 2A**). In the flavonoid biosynthesis pathway, the expression of genes Glyur000051s00003428, Glyur000336s00018105, Glyur000334s00019197, Glyur000020s00001756, Glyur000140s00011488, Glyur000044s00005205, Glyur000073s00007770, Glyur000775s00025737, Glyur002606s00033945, Glyur000959s00024504, Glyur001446s00035038, Glyur000397s00020478, Glyur001446s00035040, Glyur001216s00037797, Glyur003107s00034856, and Glyur001333s00028402 showed a significant positive correlation with the accumulation of luteolin, galangin, quercetin, myricetin, genistein, dihydromyricetin, and vitexin (**Supplementary Figure 2B**). In the flavone and flavonol biosynthesis pathway, the accumulation of isovitexin, luteolin, trifolin, quercetin, quercetin 3-O-robinobioside, myricetin, and vitexin showed a significant positive correlation with the abundance of genes Glyur000020s00001756, Glyur000775s00025737, and Glyur003107s00034856 (**Supplementary Figure 2C**).

## 4 Discussion

Na_2_SO_4_ stress induced reactive oxygen species (O_2_^−^ and H_2_O_2_) accumulation in *G. uralensis* seedlings, elevating lipid peroxidation (increased MDA and electrolyte leakage). This induced higher activities of antioxidant enzymes (POD, SOD, and CAT) (Li et al., 2023), yet these responses were insufficient to mitigate salt-induced growth inhibition, as indicated by a significant reduction in biomass. GB treatment significantly enhanced SOD, CAT, POD, and APX activities, as well as AsA content, while decreasing oxygen species (O_2_^−^ and H_2_O_2_) levels, reducing lipid peroxidation (MDA and conductivity). These changes corresponded closely to the significant upregulation of phenylpropanoid biosynthesis pathways (transcriptome) and the substantial accumulation of flavonoids and coenzyme Q_2_ (metabolome). Lignin, flavonoids, and antioxidant enzymes produced through the phenylpropanoid biosynthesis pathway are essential elements for plants to resist abiotic stress (Yao et al., 2021). Compared to CK, 19 peroxidase (POD)-encoding DEGs were enriched in S+GB+24h, with most showing significant upregulation, indicating POD as a key GB-responsive factor under salt stress. Coenzyme Q_2_, a lipophilic antioxidant, participates in mitochondrial electron transport and ROS elimination (Andrew et al., 2004). Flavonoids and flavonols act as non-enzymatic antioxidants with chelation (Andrade et al., 2018) and neutralization effects (Nuttawisit et al., 2016), synergistically enhancing ROS scavenging by antioxidant enzymes. GO enrichment confirmed that GB-induced DEGs were annotated to ‘oxidoreductase activity, acting on paired donors, with incorporation or reduction of molecular oxygen’ and ‘oxidoreductase activity, acting on paired donors, with oxidation of a pair of donors resulting in the reduction of molecular oxygen to two molecules of water’. In conclusion, GB-induced antioxidant enzyme gene expression and diverse antioxidant accumulation contributed to reduced lipid peroxidation.

Under high salinity, plants rapidly enhance intracellular osmotic potential through Na^+^, K^+^ and Ca^2+^ accumulation (Abdullakasim et al., 2018), while balancing osmotic pressure via increased proline, soluble sugars, and soluble protein concentrations (Zhang et al., 2024). Under salt stress, the contents of soluble proteins, proline, soluble sugars, and endogenous betaine in the roots and leaves of *G. uralensis* show varying degrees of increase, and the addition of GB significantly enhances this trend. Metabolomic profiling further revealed substantial accumulation of hydroxyproline, L-aspartate, and betaine in GB-treated cells, indicating that GB sustains cellular osmotic homeostasis through enhanced biosynthesis of osmoregulatory compounds. Furthermore, the enhanced BADH2 activity in roots, stems, and leaves following GB application indicates that elevated endogenous GB levels resulted not merely from exogenous GB absorption, but more critically from GB-induced transcriptional regulation. Notably, significantly higher BADH2 activity in roots versus leaves demonstrates superior endogenous GB biosynthesis capacity in root tissues. Thus, root-targeted GB application may represent an optimized strategy for efficacy enhancement. As salt ions accumulate within cells, plants detoxify through active ion excretion. *G. uralensis* employs both salt glands and stomata on abaxial leaf surfaces for this excretory function (Jiang et al., 2019). Compared with the CK, under Na_2_SO_4_ stress, exudate deposition around salt glands and stomata increased significantly, with Na^+^ secretion rate surging by 560.87%. This indicates pivotal roles of ion excretion in salt tolerance mechanisms. GB treatment further enhanced foliar ion secretion, demonstrating that exogenous GB promotes salt ion excretion to improve plant adaptation to salinity. However, high concentrations of GB (80 mM) disrupted the antioxidant, osmotic regulation, and salt secretion of licorice, which may be due to excessive GB reducing extracellular water potential, causing cytoplasmic wall separation, and interfering with the normal physiological processes of licorice (Torre-González et al., 2018).

The accumulation of lipids is beneficial for the repair of cell membranes and the stability of the intracellular environment (Renu et al., 2022). As key substrates for plant lipoxygenase, linoleic and linolenic acids are further metabolized into volatile aldehydes and jasmonates (JAs), thereby enhancing hormonal metabolism (Ren et al., 2022). These fatty acids additionally improve plant salt tolerance through modulating reactive oxygen species (ROS) levels (Ou et al., 2025). Cutin, suberin, and waxes are essential for strengthening the protective function of cell walls and reducing water loss. In this study, 6h GB treatment upregulated the expression of genes associated with fatty acid synthesis (including linoleic acid biosynthesis and general fatty acid metabolism) along with those involved in cutin, suberin, and wax biosynthesis, concurrently promoting lipid metabolite accumulation (20-Hydroxy-(5Z,8Z,11Z,14Z)eicosatetraenoic acid, 16(R)-HETE, 2,3-Dinor-8-epi-prostaglandin F2α, arachidonic acid, (+/-)12(13)-DiHOME, palmitic acid, eicosapentaenoic acid); while 24h GB treatment activated the tryptophan metabolic and brassinosteroid biosynthetic pathways. GO enrichment analysis further revealed that following 24h GB exposure, DEGs annotated within the molecular function category were significantly enriched in terms such as cell wall organization or biogenesis, cell wall modification, and external encapsulating structure organization. In summary, exogenous GB supports cell membrane integrity, enhances cell wall protection, and boosts hormone metabolism, thereby improving the tolerance of *G. uralensis* to Na_2_SO^4^ stress.

Terpenoids serve as a critical salinity defense strategy in *Glycyrrhiza* species (Shirazi et al., 2019), through their potent antioxidant activity (Wang et al., 2024) and membrane fluidity enhancement capacity (Nesterkina et al., 2018). However, studies reveal that high-level monoterpene biosynthesis—particularly volatile monoterpenes—demands substantial carbon skeletons and energy expenditure, with most compounds released into the environment. This resource-intensive mechanism demonstrates limited sustainability and is primarily effective for acute short-term stress adaptation. In our investigation, salt stress significantly upregulated monoterpenoid biosynthetic pathways, driving marked accumulation of volatile monoterpenes including carvone, perillyl alcohol, geraniol, and (-)-α-pinene. Crucially, exogenous GB application induced metabolic reprogramming: suppressing monoterpene accumulation while specifically enriching flavone and flavonol biosynthesis pathways, elevating key metabolite levels. This strategic shift demonstrates that GB redirects plant defense investment—transitioning from high-cost emergency responses (monoterpene synthesis) toward sustained antioxidant protection (flavonoid/flavonol accumulation)—thereby enhancing salinity adaptation.

The combined analysis of transcriptome and metabolome revealed the temporal regulatory characteristics of licorice response to GB under salt stress. Under salt stress, the plant hormone signaling pathway was the most active pathway after 6 h of GB treatment. As the treatment time extended to 24 h, starch and sucrose metabolism, as well as phenylpropanoid metabolism, became the main pathways regulated by GB. This indicates that licorice has undergone an adaptive transition from early signal perception to late metabolic network reconstruction in response to salt stress (Chen et al., 2023). It is worth noting that although DEGs in the starch and sucrose metabolism pathways are generally upregulated, the content of key metabolites (trehalose-6-phosphate, sucrose alpha, and alpha trehalose) is significantly reduced. This may be due to gene upregulation driving starch degradation (such as β-amylase activation) and sucrose conversion, leading to the rapid utilization of products for energy supply; In addition, GB may replace some of the osmotic regulation functions of carbohydrates to reduce excessive consumption of carbon metabolites and maintain basic energy supply. The phenylpropanoid metabolic pathway is a key defense pathway in plants in response to salt stress. Its product lignin can enhance the cell wall’s ability to resist osmotic stress, while flavonoids exert antioxidant effects by clearing ROS (Wang et al., 2021). GB treatment significantly activated phenylpropanoid metabolism and numerous downstream branching pathways after 24 h. The final products of the flavone and flavonol biosynthesis pathways, such as Isovitexin, Luteolin, Trifolin, Quercetin, Quercetin 3-O-rhamnoside, Myricetin and Vitexin, significantly increased, indicating that the synthesis of flavonols is a key mechanism for GB regulation under salt stress. Among them, luteolin, as a key metabolite, is synthesized by flavonoid 3’-monooxygenase catalysis and further modified to produce derivatives such as quercetin 3-O-sophoroside and myricetin 3-O-galactoside. Correlation analysis shows that the expression of genes Glyur000020s00001756, Glyur000775s00025737, and Glyur003107s00034856, which are speculated to encode flavonoid 3’-monooxygenase, is significantly positively correlated with the accumulation of downstream flavonol compounds. This indicates that GB drives the synthesis of luteolin and the activation of its downstream metabolic network by inducing the expression of these genes. In summary, GB significantly enhances the salt tolerance of licorice by coordinating the upstream flux allocation of phenylpropanoid metabolism pathway and downstream flavonoid synthesis. The key genes and metabolites of this pathway can serve as molecular targets for genetic improvement of salt tolerant crops or the development of exogenous regulatory strategies.

## 5 Conclusion

**Figure 14:**
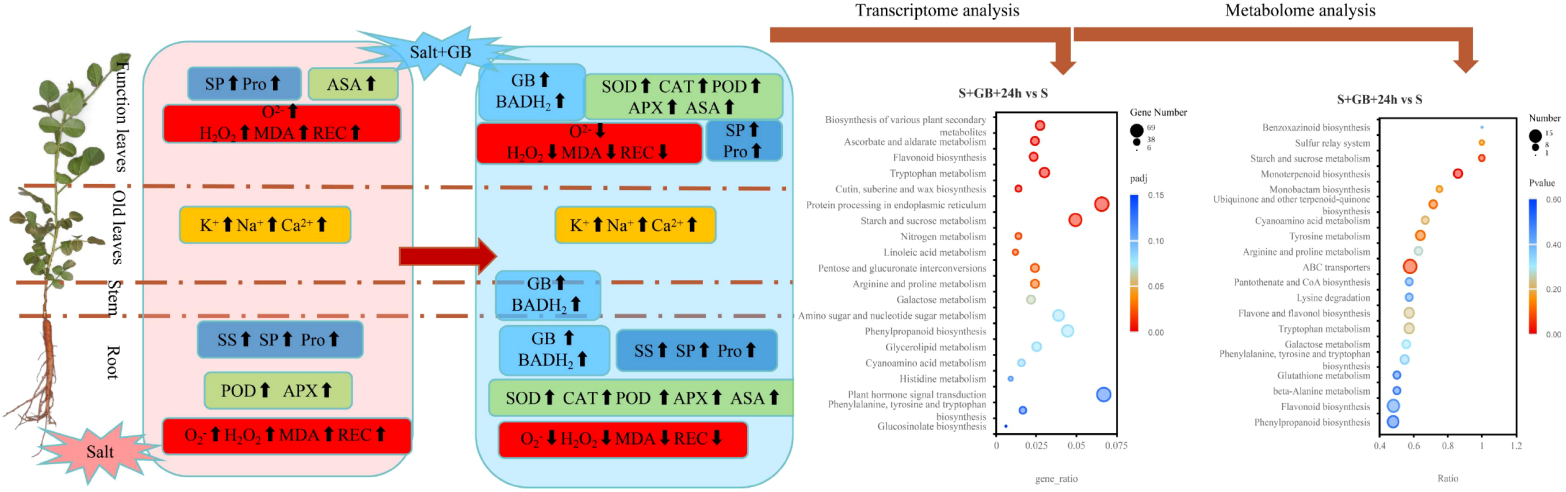
A graphical summary of the response of *G. uralensis* to glycine betaine (GB) under Na_2_SO_4_ stress. Note: Salt stress induces the accumulation of reactive oxygen species (ROS, including H_2_O_2_ and O_2_^-^) and malondialdehyde (MDA). Although it promotes the activities of antioxidant enzymes (peroxidase (POD), ascorbate peroxidase (APX) and ascorbate peroxidase (ASA)) and increases the levels of osmolytes (proline (Pro), soluble sugars (SS), soluble proteins (SP)), while moderately enhancing the secretion of K^+^, Na^+^, and Ca²^+^ ions, it ultimately leads to membrane lipid peroxidation (as indicated by elevated relative electrical conductivity (REC)). Exogenous glycine betaine (GB) enhances betaine synthesis by upregulating betaine aldehyde dehydrogenase (BADH2) activity, activates the antioxidant system (increasing the activities of superoxide dismutase (SOD), POD, catalase (CAT), APX, and ASA) to scavenge excess ROS, and significantly promotes the accumulation of osmolytes (Pro, SS, SP). It also substantially increases the rate of salt ion secretion, thereby maintaining cellular osmotic homeostasis and alleviating membrane damage (reduced REC). Bubble plots further demonstrate that GB promotes the expression of genes and the accumulation of metabolites involved in antioxidant defense, osmotic regulation, membrane protection, and signal transduction under salt stress conditions.

Na_2_SO_4_ stress induced lipid peroxidation in *G.uralensis* seedlings, suppressed the expression of most genes, disrupted the original metabolic patterns, and ultimately inhibited biomass accumulation. Exogenous GB application elevated endogenous hormone levels via enhanced tryptophan metabolism and brassinosteroid biosynthesis; induced expression of antioxidant enzyme-encoding genes (POD) and accumulation of non-enzymatic antioxidants (coenzyme Q_2_, flavonoids, and flavonols); promoted osmotic adjustment through accumulation of compatible solutes (soluble sugars, soluble proteins, proline, L-aspartic acid, and betaine); augmented leaves salt secretion capacity; improved cell membranes repair ability; redirected metabolic flux (reducing terpenoid biosynthesis). Collectively, these responses alleviated lipid peroxidation damage and increased biomass accumulation in *G. uralensis* under salt stress and supporting its potential as a low-cost, sustainable agrochemical for cultivating medicinal crops in marginal saline lands. Genes involved in phenylpropanoid metabolism, flavonoid biosynthesis, and flavone and flavonol biosynthesis are potential candidates for enhancing salt tolerance. This study elucidates the physiological and molecular mechanisms by which GB alleviates salt stress in *G.uralensis*, providing scientific evidence for improving crop salt tolerance, and enhancing yield and quality through exogenous GB application, while offering candidate genes for the breeding of salt-tolerant licorice germplasm (**Figure 14**).

## Data Availability

The data presented in the study are deposited in the NCBI repository, accession number PRJNA1293687.
