# Supplementary material for: Physiological and molecular mechanisms of glycine betaine in alleviating Na2SO4 stress in Glycyrrhiza uralensis
Source: Front Plant Sci. 2025 Nov 4;16:1667006. doi: 10.3389/fpls.2025.1667006 (PMC12674169; doi:10.3389/fpls.2025.1667006)
Supplement: Supplementary file 3 [file DataSheet3.docx]

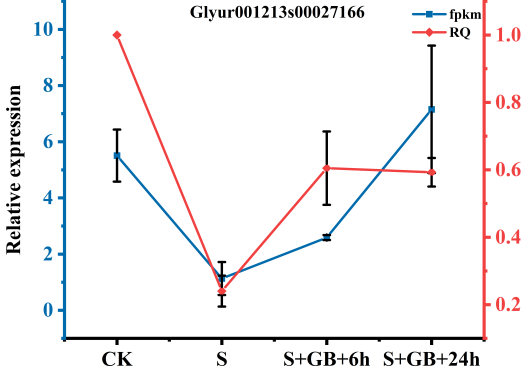


A


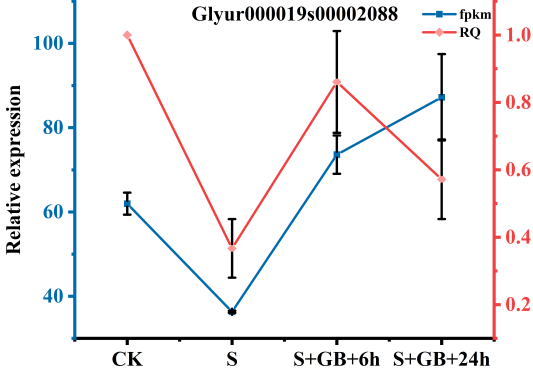


B


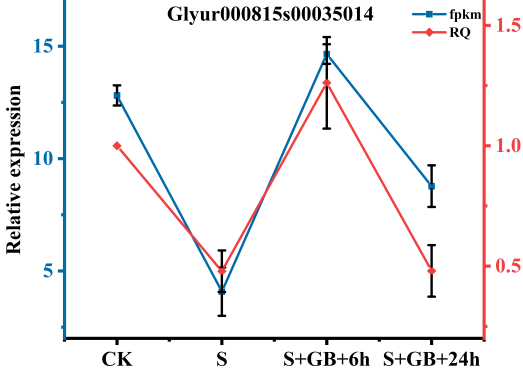


C


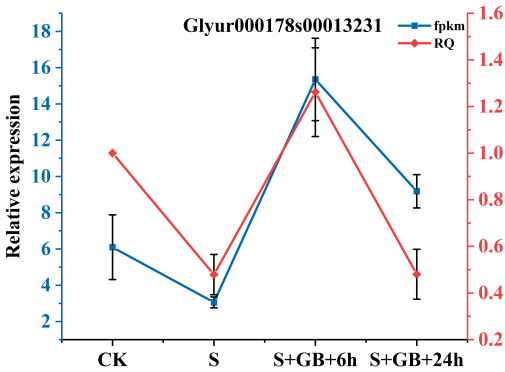


D


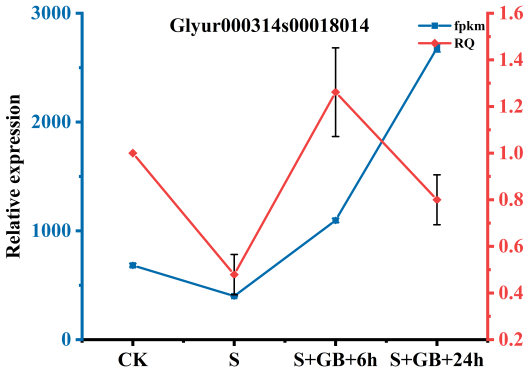


E

Supplementary Figure 1. QRT-PCR validation of differentially expressed genes Glyur001213s00027166 (A), Glyur000019s00002088 (B), Glyur000815s00035014 (C), Glyur000178s00013231 (D), Glyur000314s00018014 (E).


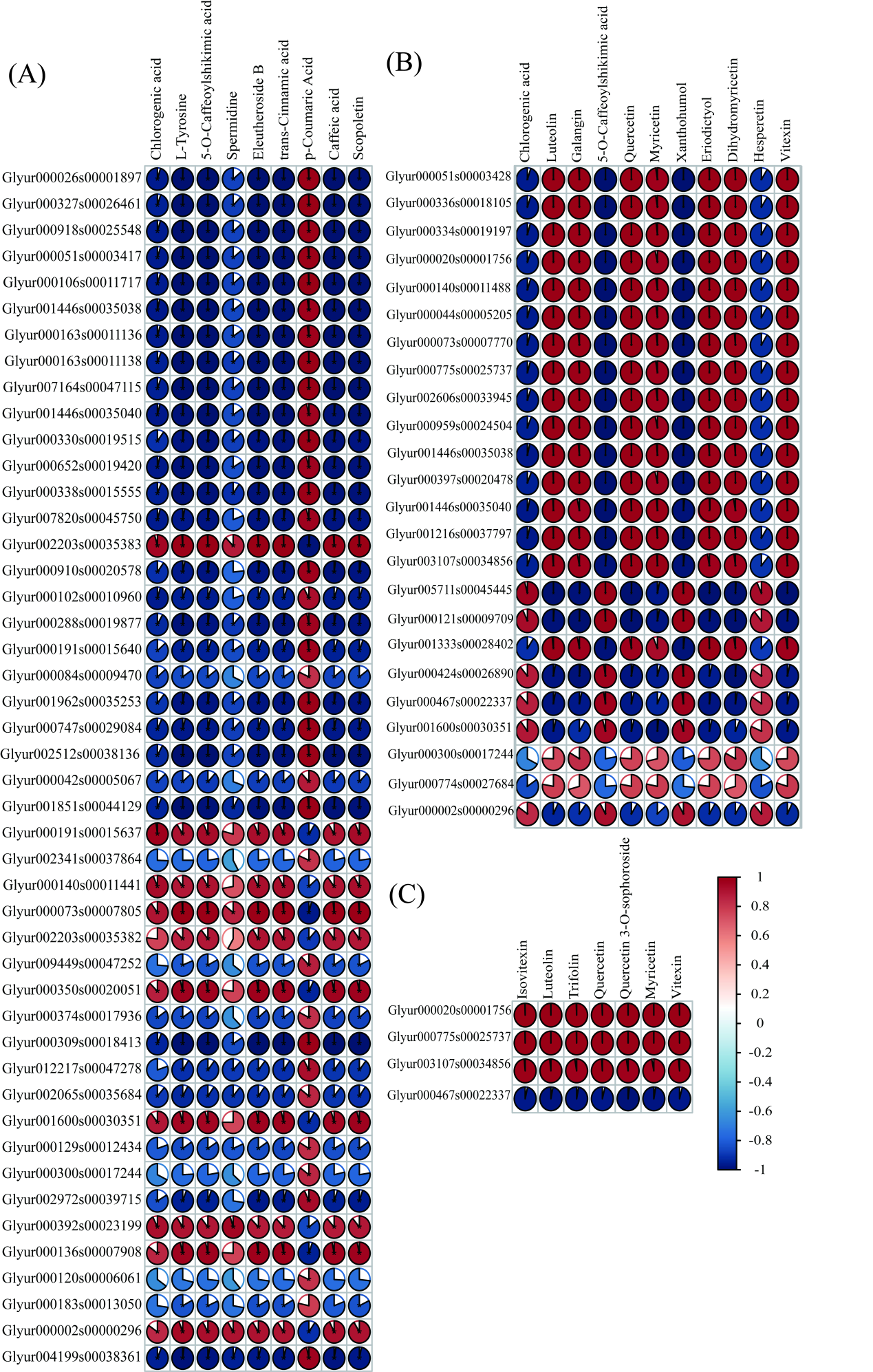


Supplementary Figure 2. Correlation analysis between DMs and DEGs in the biosynthesis of phenylpropanoids (A), flavonoid biosynthesis (B) and flavone and flavonol synthesis (C). The area of the sector represents the correlation coefficient rho, and the color represents the *P*-value, with redder colors indicating greater significance. In the result figure, parts marked with * indicate *P* ≤ 0.05.

Supplementary Table 1. Primers used for qPCR

| **Amplified gene** | **primer name** | **Primer sequence** |
| --- | --- | --- |
| Glyur001213s00027166 | 27166-F | GGAAACTCATCAGCACCACC |
|  | 27166-R | CAAGTGCTGCATCAAGGGAG |
| Glyur000019s00002088 | 02088-F | CATGATGCTCAGCGAGGTTG |
|  | 02088-R | CCTTGAACACCCTCAGCAAC |
| Glyur000815s00035014 | 35014-F | GTGGGGATAACAGCTGGGAT |
|  | 35014-R | TTGACCTGACCATACACCCC |
| Glyur000178s00013231 | 13231-F | TTTGGTGGGTTTGATGCAGG |
|  | 13231-R | AATGGGTTTGTTGGTGGACG |
| Glyur000314s00018014 | 18014-F | AGAGAGGACCGAGGAGAACT |
|  | 18014-R | GCAGCAACACAGAACACGTA |
| Actin2NEW | Actin2Forward | CCTCATGCCATCCTTCGTC |
|  | Actin2Reverse | TCTTTGCAGTCTCGAGTTCTTG |

Supplementary Table 2. Sequencing output statistics

| **Sample** | **Raw_reads** | **Raw_bases** | **Clean_reads** | **Clean_bases** | **Error_rate** | **Q20** | **Q30** | **GC_pct** |
| --- | --- | --- | --- | --- | --- | --- | --- | --- |
| CK_1 | 44758444 | 6.71G | 43291362 | 6.49G | 0.03 | 97.58 | 93.4 | 44.51 |
| CK_2 | 40422980 | 6.06G | 39327626 | 5.9G | 0.03 | 97.73 | 93.57 | 45.16 |
| CK_3 | 39577258 | 5.94G | 38415494 | 5.76G | 0.03 | 97.79 | 93.76 | 45.03 |
| S_1 | 43704466 | 6.56G | 42722238 | 6.41G | 0.03 | 97.64 | 93.41 | 45.3 |
| S_2 | 42840982 | 6.43G | 41689614 | 6.25G | 0.03 | 97.61 | 93.33 | 45.35 |
| S_3 | 42766016 | 6.41G | 41860346 | 6.28G | 0.03 | 97.95 | 94.1 | 45.37 |
| S+GB+6h_1 | 46895710 | 7.03G | 45783402 | 6.87G | 0.03 | 97.63 | 93.46 | 45.35 |
| S+GB+6h_2 | 44866340 | 6.73G | 43926704 | 6.59G | 0.03 | 97.68 | 93.49 | 45.43 |
| S+GB+6h_3 | 42073272 | 6.31G | 40862080 | 6.13G | 0.03 | 97.75 | 93.66 | 45.42 |
| S+GB+24h_1 | 46802994 | 7.02G | 45829378 | 6.87G | 0.03 | 97.42 | 93.02 | 44.56 |
| S+GB+24h_2 | 42657912 | 6.4G | 41708160 | 6.26G | 0.03 | 97.66 | 93.48 | 44.62 |
| S+GB+24h_3 | 42246468 | 6.34G | 40883670 | 6.13G | 0.03 | 97.74 | 93.7 | 44.51 |

Supplementary Table 3. Number of differentially expressed genes (DEGs) in significantly enriched pathways

| **Description** | **S vs CK** | | | **S+GB+6h vs S** | | | **S+GB+24h vs S** | | |
| --- | --- | --- | --- | --- | --- | --- | --- | --- | --- |
|  | **DEGs/all** | | **padj** | **DEGs/all** | | **padj** | **DEGs/all** | | **padj** |
|  | **up** | **down** |  | **up** | **down** |  | **up** | **down** |  |
| Zeatin biosynthesis | 3/625 | | 1.00 | 15/619 | | 0.00 | 11/1033 | | 0.62 |
|  | 2 | 1 |  | 10 | 5 |  | 6 | 5 |  |
| Plant-pathogen interaction | 42/625 | | 0.50 | 40/619 | | 0.03 | 49/1033 | | 0.00 |
|  | 19 | 23 |  | 27 | 13 |  | 26 | 23 |  |
| Pentose and glucuronate interconversions | 10/625 | | 0.73 | 14/619 | | 0.14 | 25/1033 | | 0.02 |
|  | 5 | 5 |  | 7 | 7 |  | 22 | 3 |  |
| Phenylpropanoid biosynthesis | 32/625 | | 0.05 | 33/619 | | 0.02 | 46/1033 | | 0.08 |
|  | 4 | 28 |  | 12 | 21 |  | 36 | 10 |  |
| Cutin, suberine and wax biosynthesis | 5/625 | | 0.69 | 10/619 | | 0.01 | 14/1033 | | 0.00 |
|  | 1 | 4 |  | 3 | 7 |  | 12 | 2 |  |
| Flavonoid biosynthesis | 22/625 | | 0.00 | 25/619 | | 0.00 | 24/1033 | | 0.00 |
|  | 1 | 21 |  | 3 | 22 |  | 18 | 6 |  |
| Starch and sucrose metabolism | 26/625 | | 0.50 | 30/619 | | 0.07 | 51/1033 | | 0.01 |
|  | 16 | 10 |  | 12 | 18 |  | 32 | 19 |  |
| Linoleic acid metabolism | 2/625 | | 1.00 | 5/619 | | 0.56 | 12/1033 | | 0.02 |
|  | 0 | 2 |  | 2 | 3 |  | 7 | 5 |  |
| Tryptophan metabolism | 28/625 | | 0.00 | 29/619 | | 0.00 | 31/1033 | | 0.00 |
|  | 8 | 20 |  | 14 | 15 |  | 19 | 12 |  |
| Biosynthesis of unsaturated fatty acids | 8/625 | | 0.19 | 10/619 | | 0.02 | 11/1033 | | 0.16 |
|  | 0 | 8 |  | 2 | 8 |  | 10 | 1 |  |
| Phenylalanine, tyrosine and tryptophan biosynthesis | 625 | | 1.00 | 619 | | 1.00 | 1033 | | 0.13 |
|  | 1 | 5 |  | 3 | 1 |  | 14 | 3 |  |
| Cyanoamino acid metabolism | 625 | | 0.67 | 619 | | 0.20 | 1033 | | 0.08 |
|  | 3 | 5 |  | 4 | 6 |  | 13 | 3 |  |
| Brassinosteroid biosynthesis | 625 | | 1.00 | 619 | | 0.93 | 1033 | | 0.35 |
|  | 1 | 1 |  | 3 | 2 |  | 6 | 5 |  |

Note:CK: Control group; S: Na₂SO₄ stress group; S+GB+6h: Na₂SO₄ stress + Glycine betaine treatment for 6 h group; S+GB+24h: Na₂SO₄ stress + Glycine betaine treatment for 24 h group. "up": Number of significantly upregulated DEGs; "down": Number of significantly downregulated DEGs; Format: "upregulated or down count / total DEGs "; padj: Adjusted *P*-value
